# Supplementary material for: Incidence of venous thromboembolism and bleeding in patients with malignant central nervous system neoplasm: Systematic review and meta-analysis
Source: PLoS One. 2024 Jun 20;19(6):e0304682. doi: 10.1371/journal.pone.0304682 (PMC11189257; doi:10.1371/journal.pone.0304682)
Supplement: S1 Appendix — (DOCX) [file pone.0304682.s001.docx]

S1 Appendix. REDCap file for data extraction.

|  | Identification |  |  |
| --- | --- | --- | --- |
| v.1.1 | ID | **v.1.1** |  |
| v.1.2 | Citation | **v.1.2** |  |
| v.1.3 | Year of publication  **Number** | **v.1.3** |  |
|  | **General characteristics** |  |  |
| v.2.1 | Study design  **1**-case report; **2**-cross-sectional; **3**-case-control; **4**-cohort | **v.2.1** |  |
| v.2.2 | Income classification  **1**-low income; **2**-lower-middle income; **3**-upper-middle income; **4**-high income | **v.2.2** |  |
| v.2.3 | Occurrence of VTE within six months  **0**-No; **1**-Yes | **v.2.3** |  |
| v.2.4 | Death within 12 months  **0**-No; **1**-Yes | **v.2.4** |  |
| v.2.5 | Death within 36 months  **0**-No; **1**-Yes | **v.2.5** |  |
| v.2.6 | Death within 60 months  **0**-No; **1**-Yes | **v.2.6** |  |
| v.2.7 | Presence of comorbidities  **0**-No; **1**-Yes | **v.2.7** |  |
| v.2.8 | Presence of metastasis at diagnosis  **0**-No; **1**-Yes | **v.2.8** |  |
|  | **Total** |  |  |
| v.3.1 | Total number of participants  **Number** | **v.3.1** |  |
| v.3.2 | Number of VTE  **Number** | **v.3.2** |  |
| v.3.3 | Number of bleeding events  **Number** | **v.3.3** |  |
| v.3.4 | Number of VTE plus bleeding  **Number** | **v.3.4** |  |
|  | **Male subgroup** |  |  |
| v.4.1 | Number of VTE for males  **Number** | **v.4.1** |  |
| v.4.2 | Number of bleeding events for males  **Number** | **v.4.2** |  |
| v.4.3 | Number of VTE plus bleeding for males  **Number** | **v.4.3** |  |
| v.4.4 | Number of male participants  **Number** | **v.4.4** |  |
|  | **Female subgroup** |  |  |
| v.5.1 | Number of VTE events for females  **Number** | **v.5.1** |  |
| v.5.2 | Number of bleeding events for females  **Number** | **v.5.2** |  |
| v.5.3 | Number of VTE plus bleeding for females  **Number** | **v.5.3** |  |
| v.5.4 | Number of female participants  **Number** | **v.5.4** |  |
|  | **Under 40 years old subgroup** |  |  |
| v.6.1 | Number of VTE among participants under 40 years  **Number** | **v.6.1** |  |
| v.6.2 | Number of bleeding events among participants under 40 years  **Number** | **v.6.2** |  |
| v.6.3 | Number of VTE plus bleeding among participants under 40 years  **Number** | **v.6.3** |  |
| v.6.4 | Number of participants under 40 years  **Number** | **v.6.4** |  |
|  | **40 to 60 years subgroup** |  |  |
| v.5.1 | Number of VTE among participants aged between 40 and 60 years  **Number** | **v.5.1** |  |
| v.5.2 | Number of bleeding events among participants aged between 40 and 60 years  **Number** | **v.5.2** |  |
| v.5.3 | Number of VTE plus bleeding among participants aged between 40 and 60 years  **Number** | **v.5.3** |  |
| v.5.4 | Number of participants aged between 40 and 60 years  **Number** | **v.5.4** |  |
|  | **Over 60 years subgroup** |  |  |
| v.6.1 | Number of VTE among participants over 60 years  **Number** | **v.6.1** |  |
| v.6.2 | Number of bleeding events among participants over 60 years  **Number** | **v.6.2** |  |
| v.6.3 | Number of VTE plus bleeding among participants over 60 years  **Number** | **v.6.3** |  |
| v.6.4 | Number of participants over 60 years  **Number** | **v.6.4** |  |
|  | **Gliomas** |  |  |
| v.7.1 | Number of VTE among diffuse gliomas  **Number** | **v.7.1** |  |
| v.7.2 | Number of bleeding events among diffuse gliomas  **Number** | **v.7.2** |  |
| v.7.3 | Number of VTE plus bleeding events among diffuse gliomas  **Number** | **v.7.3** |  |
| v.7.4 | Number of participants diagnosed with diffuse gliomas  **Number** | **v.7.4** |  |
|  | **GBM** |  |  |
| v.8.1 | Number of VTE among GBM  **Number** | **v.8.1** |  |
| v.8.2 | Number of bleeding events among GBM  **Number** | **v.8.2** |  |
| v.8.3 | Number of VTE plus bleeding among GBM  **Number** | **v.8.3** |  |
| v.8.4 | Number of participants diagnosed with GBM  **Number** | **v.8.4** |  |
|  | **Other tumors** |  |  |
| v.9.1 | Number of VTE among other malignant tumors  **Number** | **v.9.1** |  |
| v.9.2 | Number of bleeding events among other malignant tumors  **Number** | **v.9.2** |  |
| v.9.3 | Number of VTE plus bleeding among other malignant tumors  **Number** | **v.9.3** |  |
| v.9.4 | Number of participants diagnosed with other malignant tumors  **Number** | **v.9.4** |  |
