# Supplementary material for: Incidence of venous thromboembolism and bleeding in patients with malignant central nervous system neoplasm: Systematic review and meta-analysis
Source: PLoS One. 2024 Jun 20;19(6):e0304682. doi: 10.1371/journal.pone.0304682 (PMC11189257; doi:10.1371/journal.pone.0304682)
Supplement: S2 Appendix — (DOCX) [file pone.0304682.s002.docx]

S2 Appendix. Guidance for screening and data extraction.

| Step | Brief description | Guidance |
| --- | --- | --- |
| Screening | Based on reading titles and abstracts of identified citations, decide whether the citation has potential for inclusion | ***Rayyan***  Answer the following questions for inclusion:   1. Did the study include adults with CNS neoplasm? ***Yes, Maybe, No.*** 2. Was the type of study case report, case-control, cross-sectional or cohort study? ***Yes, No.*** 3. Did the study report VTE and/or bleeding events? ***Yes, Maybe, No.*** 4. Was the study published in English and/or Portuguese? ***Yes, No.***   In case of, at least, one answer “No” for any question above, exclude the citation. Provide a reason based on the following:   1. Population. 2. Type of study. 3. Outcomes. 4. Language.   In case of doubt, include the citation for retrieval and full text analysis. |
| Eligibility | Based on reading the full text of retrieved articles, track the articles that fulfill the eligibility criteria | After retrieval of the full text, answer the following questions for eligibility:   1. Did the study include adults with CNS malignant neoplasm? ***Yes, Maybe, No.*** 2. Was the type of study case report, case-control, cross-sectional or cohort study? ***Yes, No.*** 3. Did the study report VTE and/or bleeding events? ***Yes, Maybe, No.***   In case of, at least, one answer “No”, exclude the article and provide a reason based on the following:   1. Population. 2. Type of study. 3. Outcomes. |
| Critical appraisal | Based on applying JBI Critical Appraisal Checklist for Studies Reporting Prevalence/Incidence Data, decide for inclusion in the metanalysis | Based on full text analysis, answer the following questions:   1. Was the sample frame appropriated to address the target population? ***Yes, No, Unclear, Not applicable.*** 2. Were study participants sampled in an appropriate way? ***Yes, No, Unclear, Not applicable.*** 3. Was the sample size adequate? ***Yes, No, Unclear, Not applicable.*** 4. Were the study subjects and the setting described in detail? ***Yes, No, Unclear, Not applicable.*** 5. Was the data analysis conducted with sufficient coverage of the identified sample? ***Yes, No, Unclear, Not applicable*** 6. Were valid the methods used for the identification of the condition? ***Yes, No, Unclear, Not applicable*** 7. Was the condition measured in a standard, reliable way for all participants? ***Yes, No, Unclear, Not applicable*** 8. Was there appropriate statistical analysis? ***Yes, No, Unclear, Not applicable*** 9. Was the response rate adequate, and if not, was the low response rate managed appropriately? ***Yes, No, Unclear, Not applicable***   After completion of critical appraisal assessment, classified the study based on overall appraisal as ***Include, Exclude, Seek for further info***. |
| Data extraction | After critical appraisal assessment, extract the interesting data from the included articles | **Identification:** Author, yr  **General characteristics:**   1. Study design 2. Country 3. Setting 4. Participants 5. Sample size 6. Age group 7. Sex 8. Type/site of cancer 9. Health inequities 10. Lifestyle 11. Medical history and comorbidities 12. Surgery 13. Treatment 14. Outcomes 15. Follow-up |
|  |  | ***REDCap***   1. Type of study (*check box*) 2. Case report 3. Cross-sectional 4. Case-control 5. Cohort 6. World Bank Group country classifications (*check box*) 7. Low income 8. Lower-middle income 9. Upper-middle income 10. High income 11. Bleeding event (*check box*) 12. No 13. Yes 14. Not specified   3.1- Within 6 months (*check box*)? **No, Yes, Not specified**   1. VTE   4.1- Within 6 months (*check box*)? **No, Yes, Not specified**   1. Death within 12 months (*check box*)? **No, Yes, Not specified** 2. Death within 36 months (*check box*)? **No, Yes, Not specified** 3. Death within 60 months (*check box*)? **No, Yes, Not specified** 4. Presence of comorbidities (*check box*)? **No, Yes, Not specified** 5. Diagnosis recurrence (*check box*)? **No, Yes** 6. TOTAL 7. Total number of participants (Absolute value or 0 or 999) 8. Number of VTE (Absolute value or 0 or 999) 9. Number of bleeding events (Absolute value or 0 or 999) 10. Number of VTE plus bleeding (Absolute value or 0 or 999) 11. Male subgroup 12. Number of participants (Absolute value or 0 or 999) 13. Number of VTE (Absolute value or 0 or 999) 14. Number of bleeding events (Absolute value or 0 or 999) 15. Number of VTE plus bleeding (Absolute value or 0 or 999) 16. Female subgroup 17. Number of participants (Absolute value or 0 or 999) 18. Number of VTE (Absolute value or 0 or 999) 19. Number of bleeding events (Absolute value or 0 or 999) 20. Number of VTE plus bleeding (Absolute value or 0 or 999) 21. <40 yr subgroup 22. Number of participants (Absolute value or 0 or 999) 23. Number of VTE (Absolute value or 0 or 999) 24. Number of bleeding events (Absolute value or 0 or 999) 25. Number of VTE plus bleeding (Absolute value or 0 or 999) 26. >40, <60yr subgroup 27. Number of participants (Absolute value or 0 or 999) 28. Number of VTE (Absolute value or 0 or 999) 29. Number of bleeding events (Absolute value or 0 or 999) 30. Number of VTE plus bleeding (Absolute value or 0 or 999) 31. >60yr subgroup 32. Number of participants (Absolute value or 0 or 999) 33. Number of VTE (Absolute value or 0 or 999) 34. Number of bleeding events (Absolute value or 0 or 999) 35. Number of VTE plus bleeding (Absolute value or 0 or 999) 36. Gliomas subgroup 37. Number of participants (Absolute value or 0 or 999) 38. Number of VTE (Absolute value or 0 or 999) 39. Number of bleeding events (Absolute value or 0 or 999) 40. Number of VTE plus bleeding (Absolute value or 0 or 999) 41. GBM subgroup 42. Number of participants (Absolute value or 0 or 999) 43. Number of VTE (Absolute value or 0 or 999) 44. Number of bleeding events (Absolute value or 0 or 999) 45. Number of VTE plus bleeding (Absolute value or 0 or 999) 46. Other tumors 47. Number of participants (Absolute value or 0 or 999) 48. Number of VTE (Absolute value or 0 or 999) 49. Number of bleeding events (Absolute value or 0 or 999) 50. Number of VTE plus bleeding (Absolute value or 0 or 999) |
