# Supplementary figures and images for: Incidence of venous thromboembolism and bleeding in patients with malignant central nervous system neoplasm: Systematic review and meta-analysis
Source: PLoS One. 2024 Jun 20;19(6):e0304682. doi: 10.1371/journal.pone.0304682 (PMC11189257; doi:10.1371/journal.pone.0304682)

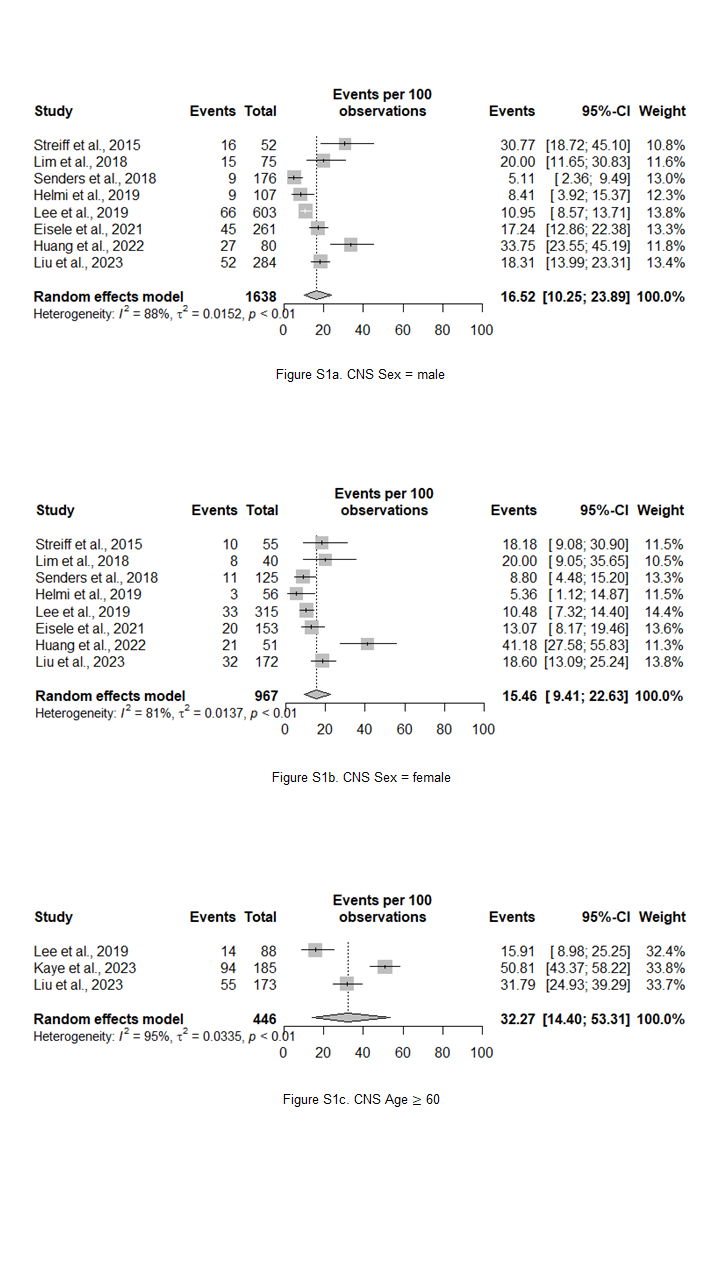

Supplement: S1 File — (ZIP) [file pone.0304682.s004.zip › S1-forests/S1-forest_a_b_c.tif]

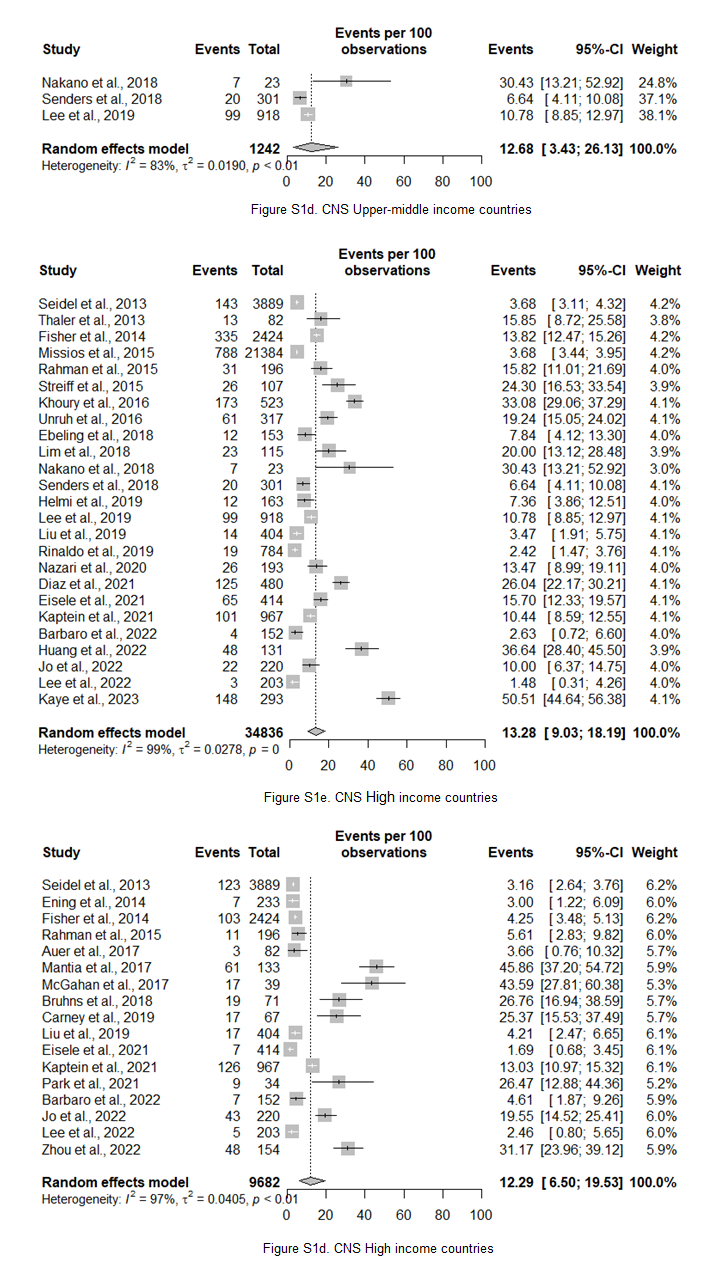

Supplement: S1 File — (ZIP) [file pone.0304682.s004.zip › S1-forests/S1-forest_d_e_f.tif]

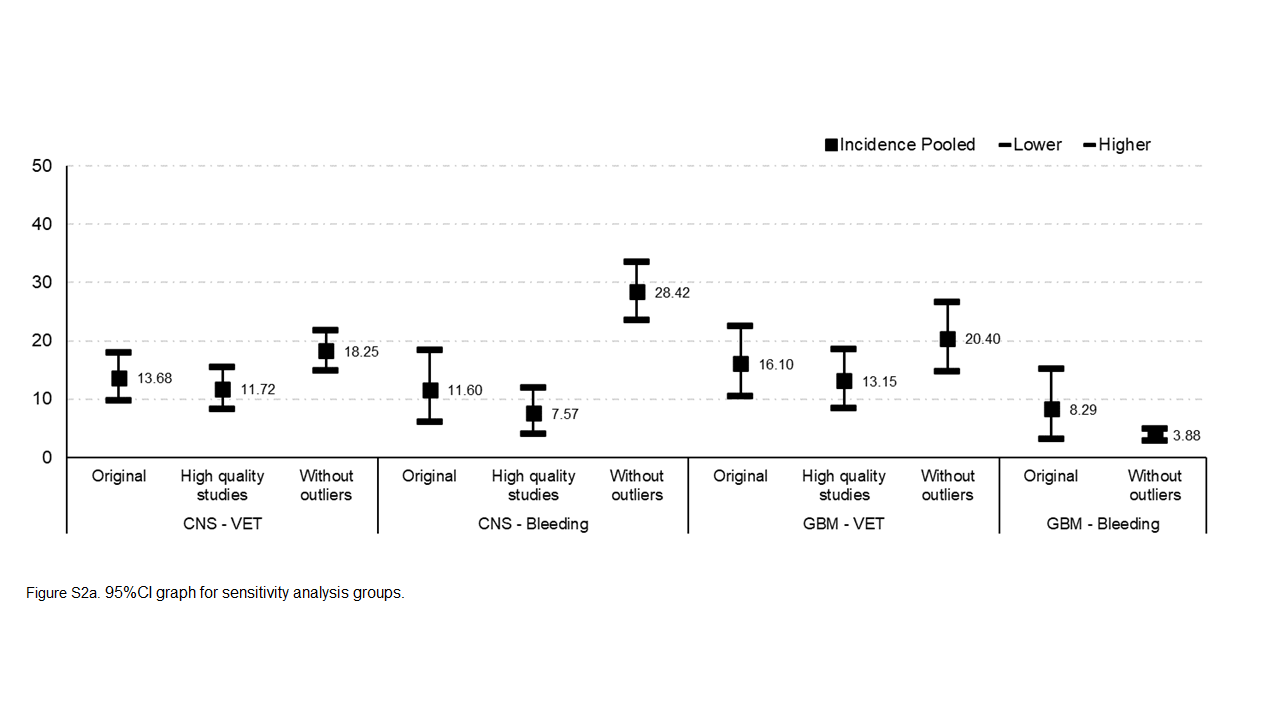

Supplement: S2 File — a-f. Forest plots of venous thromboembolism and bleeding according subgroup analysis. (ZIP) [file pone.0304682.s005.zip › S2-forest_funnel_CIgraph/CI95-distribution-studies.tif]

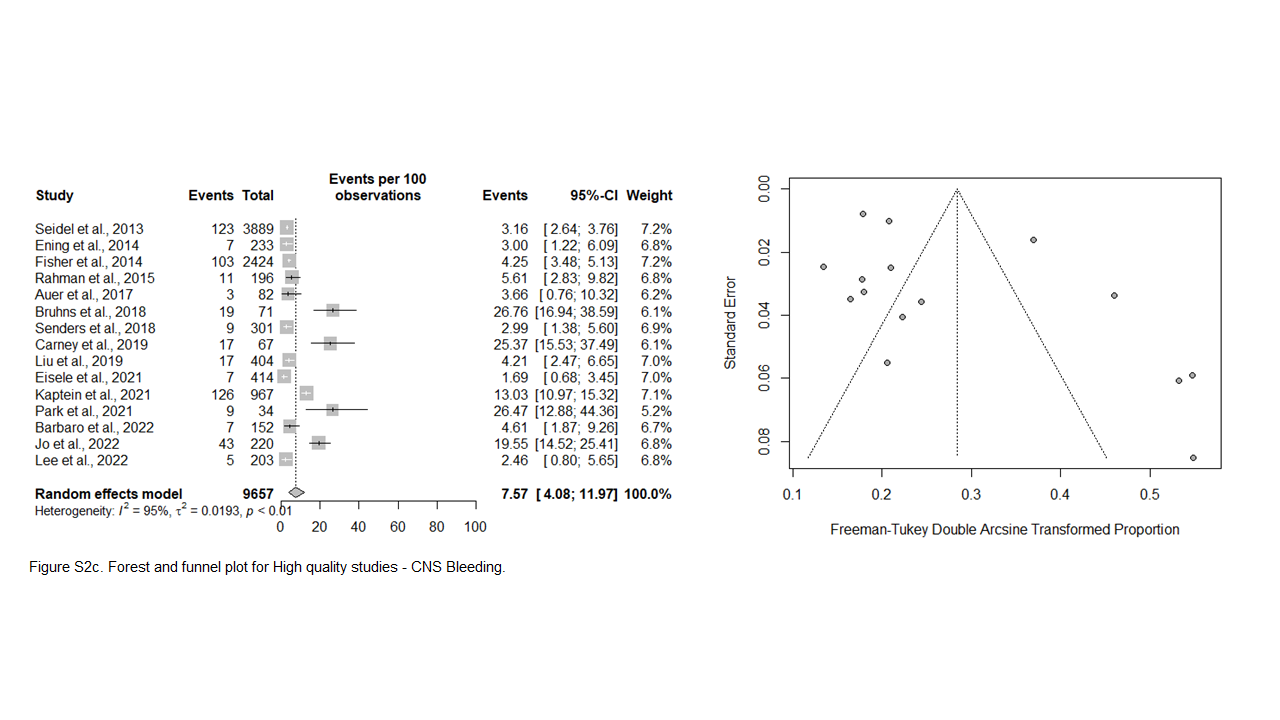

Supplement: S2 File — a-f. Forest plots of venous thromboembolism and bleeding according subgroup analysis. (ZIP) [file pone.0304682.s005.zip › S2-forest_funnel_CIgraph/High_quality_studies/CNS_Bleeding.TIF]

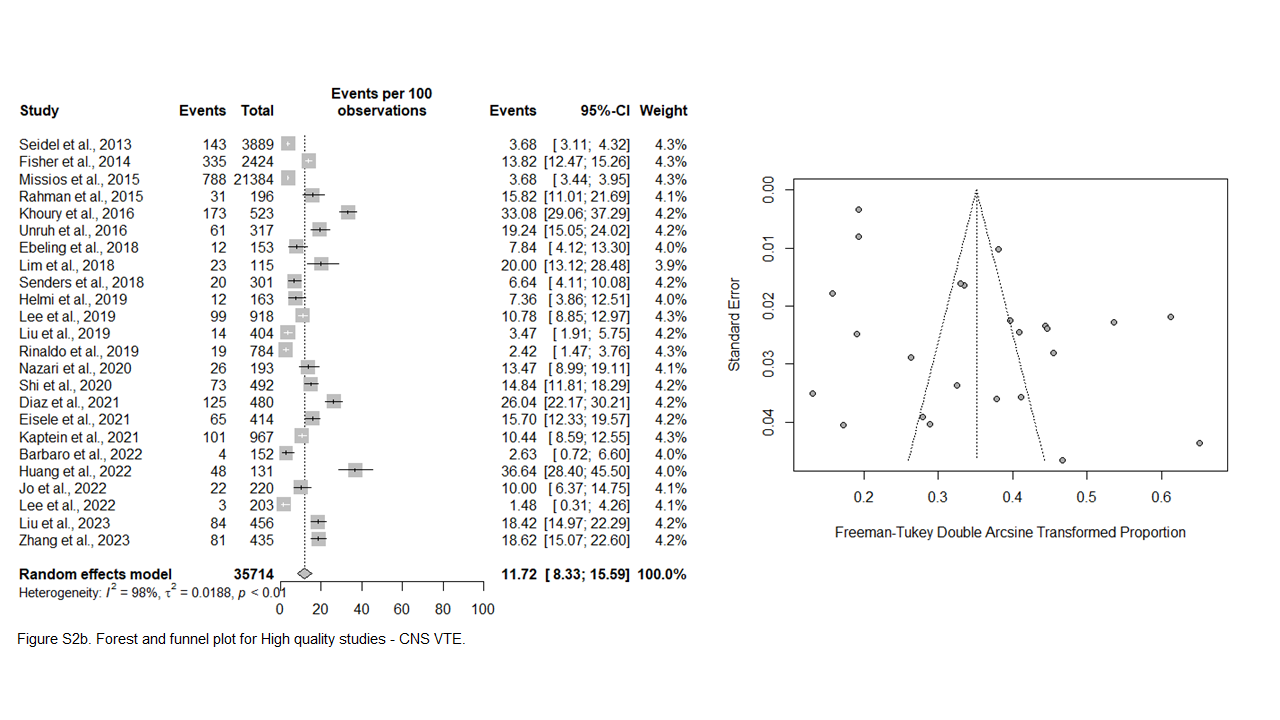

Supplement: S2 File — a-f. Forest plots of venous thromboembolism and bleeding according subgroup analysis. (ZIP) [file pone.0304682.s005.zip › S2-forest_funnel_CIgraph/High_quality_studies/CNS_VTE.TIF]

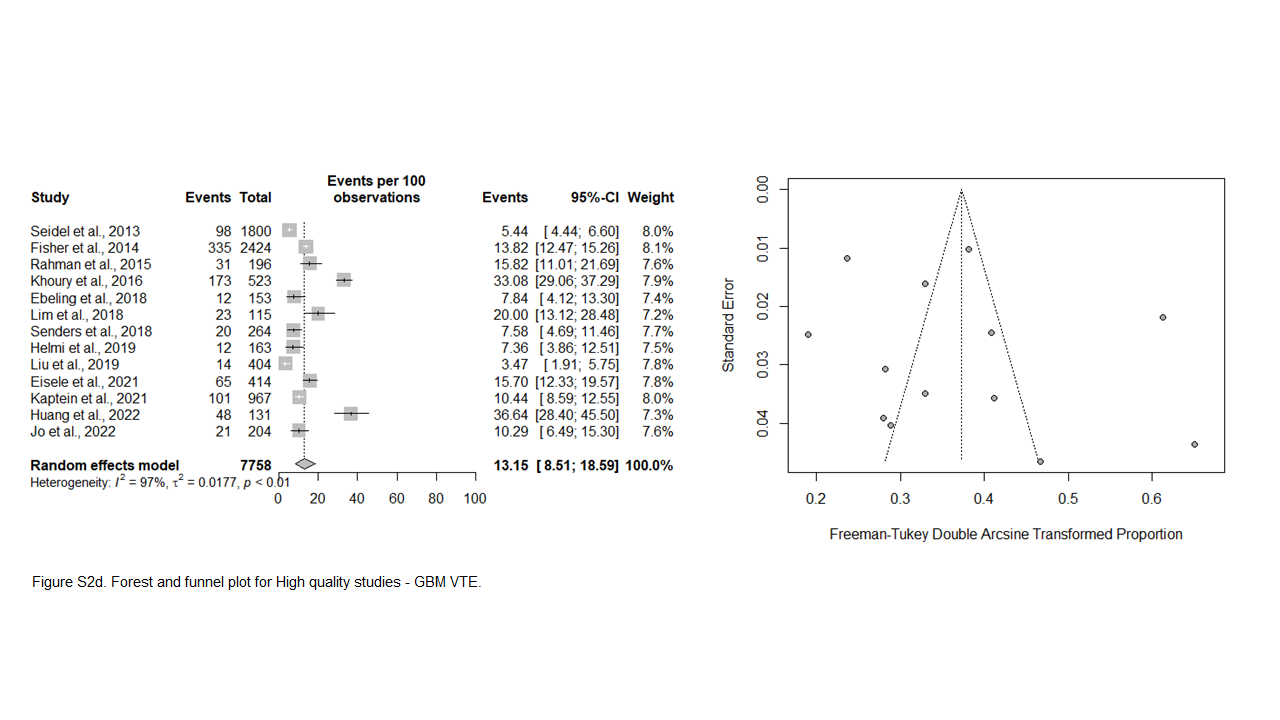

Supplement: S2 File — a-f. Forest plots of venous thromboembolism and bleeding according subgroup analysis. (ZIP) [file pone.0304682.s005.zip › S2-forest_funnel_CIgraph/High_quality_studies/GBM_VTE.TIF]

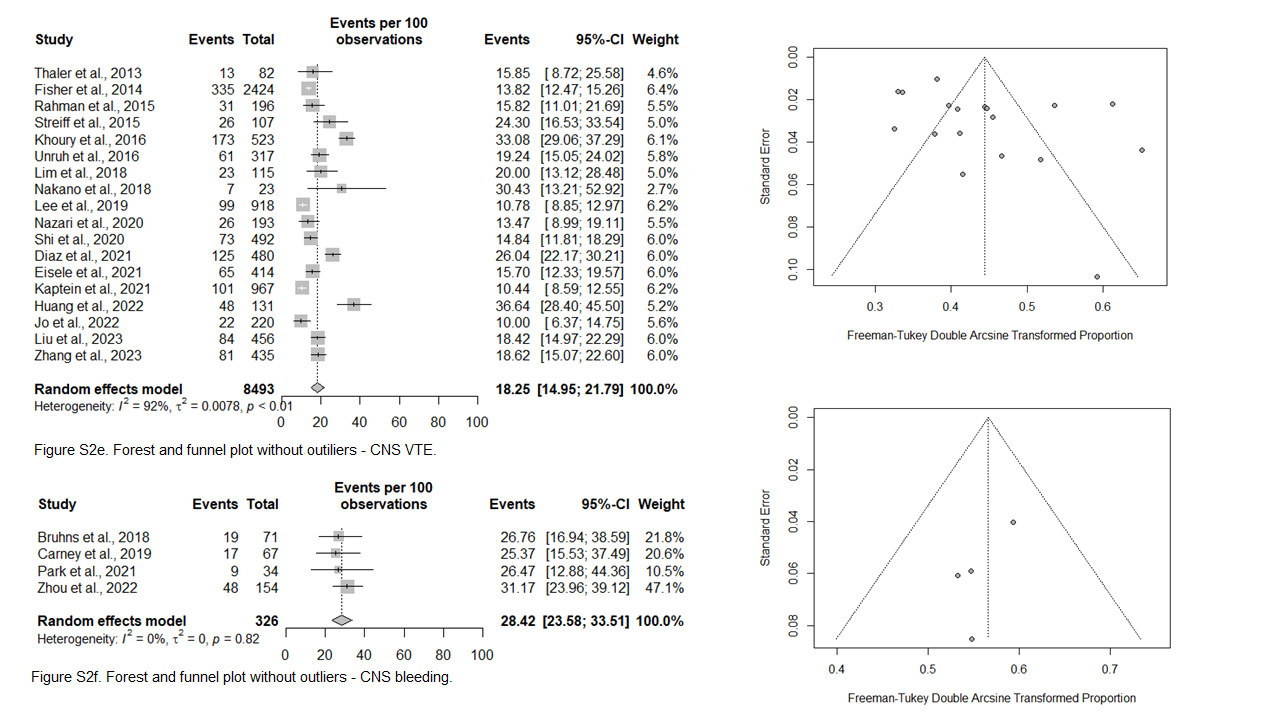

Supplement: S2 File — a-f. Forest plots of venous thromboembolism and bleeding according subgroup analysis. (ZIP) [file pone.0304682.s005.zip › S2-forest_funnel_CIgraph/Withou_outilers/CNS-VTE-Bleeding.TIF]

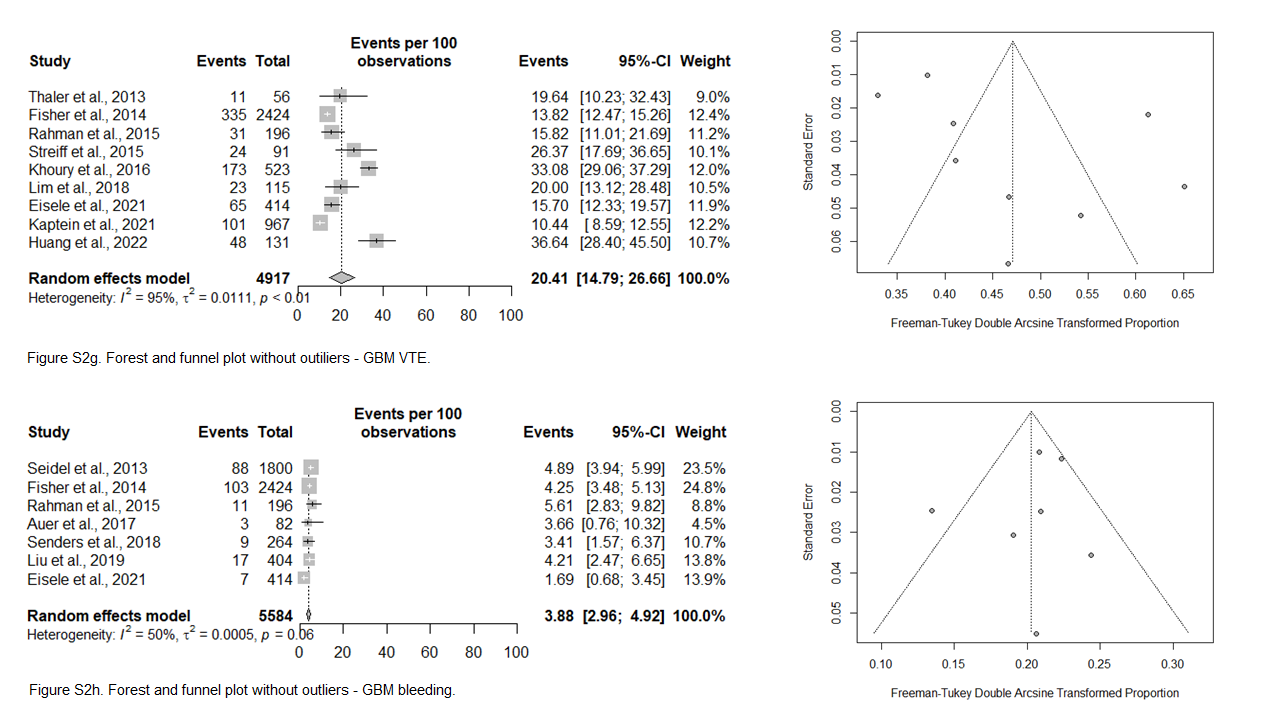

Supplement: S2 File — a-f. Forest plots of venous thromboembolism and bleeding according subgroup analysis. (ZIP) [file pone.0304682.s005.zip › S2-forest_funnel_CIgraph/Withou_outilers/GBM-VTE-Bleeding.TIF]
