# Supplementary material for: Incidence of venous thromboembolism and bleeding in patients with malignant central nervous system neoplasm: Systematic review and meta-analysis
Source: PLoS One. 2024 Jun 20;19(6):e0304682. doi: 10.1371/journal.pone.0304682 (PMC11189257; doi:10.1371/journal.pone.0304682)
Supplement: S1 Table — (DOCX) [file pone.0304682.s006.docx]

S1 Table. Search strategy according to electronic databases.

| Database | PubMed | Date | 30/06 to 03/07 |
| --- | --- | --- | --- |
| #1 | (central nervous system neoplasms[MeSH Terms]) OR (CNS neoplasm) OR (CNS neoplasms) OR (central nervous system neoplasm) OR (central nervous system neoplasms, primary[MeSH Terms]) OR (central nervous system tumor)) OR (central nervous system tumors[MeSH Terms]) OR (neoplasm, CNS)) OR (neoplasms, CNS) OR (neoplasms, central nervous system)) OR (primary central nervous system neoplasm) OR (primary central nervous system neoplasms[MeSH Terms])) OR (tumors central nervous system[MeSH Terms]) OR (brain neoplasms[MeSH Terms]) OR (brain cancer[MeSH Terms])) OR (brain cancers[MeSH Terms])) OR (brain malignant neoplasm[MeSH Terms])) OR (brain malignant neoplasms[MeSH Terms])) OR (brain neoplasm)) OR (brain neoplasm, malignant[MeSH Terms])) OR (brain neoplasm, primary[MeSH Terms])) OR (brain neoplasms, malignant[MeSH Terms])) OR (brain neoplasms, malignant, primary[MeSH Terms])) OR (brain neoplasms, primary[MeSH Terms])) OR (brain neoplasms, primary malignant[MeSH Terms])) OR (brain tumor[MeSH Terms])) OR (brain tumor, primary[MeSH Terms])) OR (brain tumor, recurrent[MeSH Terms])) OR (brain tumors[MeSH Terms])) OR (brain tumors, primary[MeSH Terms])) OR (brain tumors, recurrent[MeSH Terms])) OR (cancer of brain[MeSH Terms])) OR (cancer of the brain[MeSH Terms])) OR (cancer, brain[MeSH Terms])) OR (cancers, brain[MeSH Terms])) OR (intracranial neoplasm[MeSH Terms])) OR (intracranial neoplasms[MeSH Terms])) OR (malignant brain neoplasm[MeSH Terms])) OR (malignant brain neoplasms[MeSH Terms])) OR (malignant neoplasm, brain[MeSH Terms])) OR (malignant neoplasms, brain[MeSH Terms])) OR (malignant primary brain neoplasms[MeSH Terms])) OR (malignant primary brain tumors[MeSH Terms])) OR (neoplasm, brain[MeSH Terms])) OR (neoplasm, intracranial[MeSH Terms])) OR (neoplasms, brain[MeSH Terms])) OR (neoplasms, brain, malignant[MeSH Terms])) OR (neoplasms, brain, primary[MeSH Terms])) OR (neoplasms, intracranial[MeSH Terms])) OR (primary brain neoplasm[MeSH Terms])) OR (primary brain neoplasms[MeSH Terms])) OR (primary brain tumor[MeSH Terms])) OR (primary brain tumors[MeSH Terms])) OR (primary malignant brain neoplasms[MeSH Terms])) OR (primary malignant brain tumors[MeSH Terms])) OR (recurrent brain tumor[MeSH Terms])) OR (recurrent brain tumors[MeSH Terms])) OR (tumor, brain[MeSH Terms])) OR (tumors, brain[MeSH Terms]))) OR (((((((((((((((((((((((((((((((cerebral ventricle neoplasm[MeSH Terms]) OR (brain ventricular neoplasm[MeSH Terms])) OR (brain ventricular neoplasms[MeSH Terms])) OR (brain ventricular tumor[MeSH Terms])) OR (brain ventricular tumors[MeSH Terms])) OR (cerebral ventricle neoplasm[MeSH Terms])) OR (cerebral ventricle tumor[MeSH Terms])) OR (cerebral ventricle tumors[MeSH Terms])) OR (cerebroventricular neoplasm[MeSH Terms])) OR (cerebroventricular neoplasms[MeSH Terms])) OR (intraventricular neoplasm[MeSH Terms])) OR (intraventricular neoplasms[MeSH Terms])) OR (neoplasm, brain ventricular[MeSH Terms])) OR (neoplasm, cerebral ventricle[MeSH Terms])) OR (neoplasm, cerebroventricular[MeSH Terms])) OR (neoplasm, intraventricular[MeSH Terms])) OR (neoplasms, brain ventricular[MeSH Terms])) OR (neoplasms, cerebral ventricle[MeSH Terms])) OR (neoplasms, cerebroventricular[MeSH Terms])) OR (neoplasms, intraventricular[MeSH Terms])) OR (neoplasms, ventricular, brain[MeSH Terms])) OR (tumor, brain ventricular[MeSH Terms])) OR (tumor, cerebral ventricle[MeSH Terms])) OR (tumors, brain ventricular[MeSH Terms])) OR (tumors, cerebral ventricle[MeSH Terms])) OR (ventricle tumor, cerebral[MeSH Terms])) OR (ventricle tumors, cerebral[MeSH Terms])) OR (ventricular neoplasm, brain[MeSH Terms])) OR (ventricular neoplasms, brain[MeSH Terms])) OR (ventricular tumor, brain[MeSH Terms])) OR (ventricular tumors, brain[MeSH Terms]))) OR ((((((((((((((((astroblastoma[MeSH Terms]) OR (astroblastomas[MeSH Terms])) OR (ependymoastrocytoma[MeSH Terms])) OR (ependymoastrocytomas[MeSH Terms])) OR (gliomatosis cerebri[MeSH Terms])) OR (neoplasm, neuroepithelial[MeSH Terms])) OR (neuroepithelial neoplasm[MeSH Terms])) OR (neuroepithelial neoplasms[MeSH Terms])) OR (neuroepithelial tumor[MeSH Terms])) OR (neuroepithelial tumors[MeSH Terms])) OR (polar spongioblastoma[MeSH Terms])) OR (polar spongioblastomas[MeSH Terms])) OR (spongioblastoma, polar[MeSH Terms])) OR (spongioblastomas, polar[MeSH Terms])) OR (tumor, neuroepithelial[MeSH Terms])) OR (tumors, neuroepithelial[MeSH Terms]))) OR ((((((((((((((((((((((((neuroectodermal tumors, primitive[MeSH Terms]) OR (cerebral primitive neuroectodermal tumor[MeSH Terms])) OR (ependymoblastoma[MeSH Terms])) OR (ependymoblastomas[MeSH Terms])) OR (neoplasm, primitive neuroepithelial[MeSH Terms])) OR (neoplasms, primitive neuroepithelial[MeSH Terms])) OR (neuroectodermal tumor, primitive[MeSH Terms])) OR (neuroepithelial neoplasm, primitive[MeSH Terms])) OR (neuroepithelial neoplasm, primitive[MeSH Terms])) OR (neuroepithelial neoplasms, primitive[MeSH Terms])) OR (neuroepithelial tumor, primitive[MeSH Terms])) OR (neuroepithelial tumors, primitive[MeSH Terms])) OR (primitive neuroectodermal tumor[MeSH Terms])) OR (primitive neuroectodermal tumors[MeSH Terms])) OR (primitive neuroepithelial neoplasm[MeSH Terms])) OR (primitive neuroepithelial neoplasms[MeSH Terms])) OR (primitive neuroepithelial tumor[MeSH Terms])) OR (primitive neuroepithelial tumors[MeSH Terms])) OR (spongioblastoma[MeSH Terms])) OR (spongioblastomas[MeSH Terms])) OR (tumor, primitive neuroectodermal[MeSH Terms])) OR (tumor, primitive neuroepithelial[MeSH Terms])) OR (tumors, primitive neuroectodermal[MeSH Terms])) OR (tumors, primitive neuroepithelial[MeSH Terms]))) OR ((((((((((((((((((((((((((((((((meningeal neoplasms[MeSH Terms]) OR (cancer, meningeal[MeSH Terms])) OR (cancers, meningeal[MeSH Terms])) OR (intracranial meningeal neoplasm[MeSH Terms])) OR (intracranial meningeal neoplasms[MeSH Terms])) OR (leptomeningeal neoplasm[MeSH Terms])) OR (leptomeningeal neoplasms[MeSH Terms])) OR (meningeal cancer[MeSH Terms])) OR (meningeal cancers[MeSH Terms])) OR (meningeal neoplasm[MeSH Terms])) OR (meningeal neoplasm, intracranial[MeSH Terms])) OR (meningeal neoplasm, malignant[MeSH Terms])) OR (meningeal neoplasm, spinal[MeSH Terms])) OR (meningeal neoplasms, intracranial[MeSH Terms])) OR (meningeal neoplasms, malignant[MeSH Terms])) OR (meningeal neoplasms, spinal[MeSH Terms])) OR (meningeal tumor[MeSH Terms])) OR (meningeal tumors[MeSH Terms])) OR (neoplasm, intracranial meningeal[MeSH Terms])) OR (neoplasm, leptomeningeal[MeSH Terms])) OR (neoplasm, malignant meningeal[MeSH Terms])) OR (neoplasm, meningeal[MeSH Terms])) OR (neoplasm, spinal meningeal[MeSH Terms])) OR (neoplasms, intracranial meningeal[MeSH Terms])) OR (neoplasms, leptomeningeal[MeSH Terms])) OR (neoplasms, malignant meningeal[MeSH Terms])) OR (neoplasms, meningeal[MeSH Terms])) OR (neoplasms, spinal meningeal[MeSH Terms])) OR (spinal meningeal neoplasm[MeSH Terms])) OR (spinal meningeal neoplasms[MeSH Terms])) OR (tumor, meningeal[MeSH Terms])) OR (tumors, meningeal[MeSH Terms]))) OR ((((((((((((((glioma[MeSH Terms]) OR (glial cell tumor[MeSH Terms])) OR (glial cell tumors[MeSH Terms])) OR (glioma, malignant[MeSH Terms])) OR (glioma, mixed[MeSH Terms])) OR (gliomas[MeSH Terms])) OR (gliomas, malignant[MeSH Terms])) OR (gliomas, mixed[MeSH Terms])) OR (malignant glioma[MeSH Terms])) OR (malignant gliomas[MeSH Terms])) OR (mixed glioma[MeSH Terms])) OR (mixed gliomas[MeSH Terms])) OR (tumor, glial cell[MeSH Terms])) OR (tumors, glial cell[MeSH Terms]))) OR ((((((((((ganglioglioma[MeSH Terms]) OR (ganglioglioma, intracranial[MeSH Terms])) OR (ganglioglioma, malignant[MeSH Terms])) OR (gangliogliomas[MeSH Terms])) OR (gangliogliomas, intracranial[MeSH Terms])) OR (gangliogliomas, malignant[MeSH Terms])) OR (intracranial ganglioglioma[MeSH Terms])) OR (intracranial gangliogliomas[MeSH Terms])) OR (malignant ganglioglioma[MeSH Terms])) OR (malignant gangliogliomas[MeSH Terms]))) OR (((((((((((((((((((((((((((((((((((((((((((astrocytoma[MeSH Terms]) OR (anaplastic astrocytoma[MeSH Terms])) OR (anaplastic astrocytomas[MeSH Terms])) OR (astrocytic glioma[MeSH Terms])) OR (astrocytic gliomas[MeSH Terms])) OR (astrocytoma, anaplastic[MeSH Terms])) OR (astrocytoma, cerebral[MeSH Terms])) OR (astrocytoma, fibrillary[MeSH Terms])) OR (astrocytoma, gemistocytic[MeSH Terms])) OR (astrocytoma, intracranial[MeSH Terms])) OR (astrocytoma, pilocytic[MeSH Terms])) OR (astrocytoma, protoplasmic[MeSH Terms])) OR (astrocytoma, subependymal[MeSH Terms])) OR (astrocytomas[MeSH Terms])) OR (astrocytomas, anaplastic[MeSH Terms])) OR (astrocytomas, cerebral[MeSH Terms])) OR (astrocytomas, fibrillary[MeSH Terms])) OR (astrocytomas, gemistocytic[MeSH Terms])) OR (astrocytomas, intracranial[MeSH Terms])) OR (astrocytomas, pilocytic[MeSH Terms])) OR (astrocytomas, protoplasmic[MeSH Terms])) OR (astroglioma[MeSH Terms])) OR (astrogliomas[MeSH Terms])) OR (cerebral astrocytoma[MeSH Terms])) OR (cerebral astrocytomas[MeSH Terms])) OR (fibrillary astrocytoma[MeSH Terms])) OR (fibrillary astrocytomas[MeSH Terms])) OR (gemistocytic astrocytoma[MeSH Terms])) OR (gemistocytic astrocytomas[MeSH Terms])) OR (glioma, astrocytic[MeSH Terms])) OR (gliomas, astrocytic[MeSH Terms])) OR (intracranial astrocytoma[MeSH Terms])) OR (intracranial astrocytomas[MeSH Terms])) OR (mixed oligoastrocytoma[MeSH Terms])) OR (mixed oligoastrocytomas[MeSH Terms])) OR (oligoastrocytoma, mixed[MeSH Terms])) OR (oligoastrocytomas, mixed[MeSH Terms])) OR (pilocytic astrocytoma[MeSH Terms])) OR (pilocytic astrocytomas[MeSH Terms])) OR (pleomorphic xanthoastrocytomas)) OR (protoplasmic astrocytoma[MeSH Terms])) OR (protoplasmic astrocytomas[MeSH Terms])) OR (subependymal giant cell astrocytoma[MeSH Terms]))) OR (((((((glioblastoma[MeSH Terms]) OR (giant cell glioblastoma[MeSH Terms])) OR (giant cell glioblastomas[MeSH Terms])) OR (glioblastoma, giant cell[MeSH Terms])) OR (glioblastoma multiforme[MeSH Terms])) OR (glioblastomas[MeSH Terms])) OR (glioblastomas, giant cell[MeSH Terms]))) OR ((((((((((((((((ependymoma[MeSH Terms]) OR (anaplastic ependymoma[MeSH Terms])) OR (anaplastic ependymomas[MeSH Terms])) OR (cellular ependymoma[MeSH Terms])) OR (clear cell ependymoma[MeSH Terms])) OR (ependymoma, anaplastic[MeSH Terms])) OR (ependymoma, myxopapillary[MeSH Terms])) OR (ependymoma, papillary[MeSH Terms])) OR (ependymomas[MeSH Terms])) OR (ependymomas, anaplastic[MeSH Terms])) OR (ependymomas, myxopapillary[MeSH Terms])) OR (ependymomas, papillary[MeSH Terms])) OR (myxopapillary ependymoma[MeSH Terms])) OR (myxopapillary ependymomas[MeSH Terms])) OR (papillary ependymoma[MeSH Terms])) OR (papillary ependymomas[MeSH Terms]))) OR ((((((((((((((((oligodendroglioma[MeSH Terms]) OR (adult oligodendroglioma[MeSH Terms])) OR (adult oligodendrogliomas[MeSH Terms])) OR (anaplastic oligodendroglioma[MeSH Terms])) OR (anaplastic oligodendrogliomas[MeSH Terms])) OR (mixed oligodendroglioma astrocytoma[MeSH Terms])) OR (mixed oligodendroglioma ependymoma[MeSH Terms])) OR (oligodendroblastoma[MeSH Terms])) OR (oligodendroblastomas[MeSH Terms])) OR (oligodendroglioma, adult[MeSH Terms])) OR (oligodendroglioma, anaplastic[MeSH Terms])) OR (oligodendroglioma, well differentiated[MeSH Terms])) OR (oligodendrogliomas[MeSH Terms])) OR (oligodendrogliomas, adult[MeSH Terms])) OR (oligodendrogliomas, anaplastic[MeSH Terms])) OR (well diferentiated oligodendroglioma[MeSH Terms]))) OR ((((((((((((((((((((glioma, subependymal[MeSH Terms]) OR (adult subependymal astrocytoma[MeSH Terms])) OR (adult subependymal astrocytomas[MeSH Terms])) OR (astrocytoma, adult subependymal[MeSH Terms])) OR (astrocytoma, subependymal[MeSH Terms])) OR (astrocytomas, subependymal[MeSH Terms])) OR (astrocytomas, adult subependymal[MeSH Terms])) OR (gliomas, subependymal[MeSH Terms])) OR (glioses, subependymal[MeSH Terms])) OR (gliosis, subependymal[MeSH Terms])) OR (subependymal astrocytoma[MeSH Terms])) OR (subependymal astrocytoma, adult[MeSH Terms])) OR (subependymal astrocytomas[MeSH Terms])) OR (subependymal astrocytomas, adult[MeSH Terms])) OR (subependymal glioma[MeSH Terms])) OR (subependymal gliomas[MeSH Terms])) OR (subependymal glioses[MeSH Terms])) OR (subependymal gliosis[MeSH Terms])) OR (subependymoma[MeSH Terms])) OR (subependymomas[MeSH Terms]))) OR ((malignant meningioma[MeSH Terms]) OR (malignant meningiomas[MeSH Terms])))) | | |
| #2 | **(((((((((((((((((((((((((((((((((((((cerebral hemorrhage[MeSH Terms]) OR (cerebrum hemorrhage[MeSH Terms])) OR (cerebrum hemorrhages[MeSH Terms])) OR (cerebral parenchymal hemorrhage[MeSH Terms])) OR (brain hemorrhage, cerebral[MeSH Terms])) OR (brain hemorrhages, cerebral[MeSH Terms])) OR (cerebral brain hemorrhage[MeSH Terms])) OR (cerebral brain hemorrhages[MeSH Terms])) OR (cerebral hemorrhages[MeSH Terms])) OR (cerebral parenchymal hemorrhage[MeSH Terms])) OR (cerebral parenchymal hemorrhages[MeSH Terms])) OR (cerebrum hemorrhage[MeSH Terms])) OR (cerebrum hemorrhages[MeSH Terms])) OR (hemorrhage, cerebral[MeSH Terms])) OR (hemorrhage, cerebral brain[MeSH Terms])) OR (hemorrhage, cerebral parenchymal[MeSH Terms])) OR (hemorrhage, cerebrum[MeSH Terms])) OR (hemorrhage, intracerebral[MeSH Terms])) OR (hemorrhages, cerebral[MeSH Terms])) OR (hemorrhages, cerebral brain[MeSH Terms])) OR (hemorrhages, cerebral parenchymal[MeSH Terms])) OR (hemorrhages, cerebrum[MeSH Terms])) OR (hemorrhages, intracerebral[MeSH Terms])) OR (intracerebral hemorrhage[MeSH Terms])) OR (intracerebral hemorrhages[MeSH Terms])) OR (parenchymal hemorrhage, cerebral[MeSH Terms])) OR (parenchymal hemorrhages, cerebral[MeSH Terms])) OR ((((((((((thrombosis) OR (venous thrombosis[MeSH Terms])) OR (thrombosis, deep vein[MeSH Terms])) OR (thrombosis, deep venous[MeSH Terms])) OR (thrombosis, venous[MeSH Terms])) OR (vein thromboses, deep[MeSH Terms])) OR (vein thrombosis, deep[MeSH Terms])) OR (venous thromboses[MeSH Terms])) OR (venous thromboses, deep[MeSH Terms])) OR (venous thrombosis, deep[MeSH Terms]))) OR (((((((((((((((((((intracranial thrombosis[MeSH Terms]) OR (brain thromboses[MeSH Terms])) OR (brain thrombosis[MeSH Terms])) OR (brain thrombus[MeSH Terms])) OR (cerebral thromboses[MeSH Terms])) OR (cerebral thrombosis[MeSH Terms])) OR (cerebral thrombus[MeSH Terms])) OR (intracranial thromboses[MeSH Terms])) OR (intracranial thrombus[MeSH Terms])) OR (thromboses, brain[MeSH Terms])) OR (thromboses, cerebral[MeSH Terms])) OR (thromboses, cerebral[MeSH Terms])) OR (thromboses, intracranial[MeSH Terms])) OR (thrombosis, brain[MeSH Terms])) OR (thrombosis, cerebral[MeSH Terms])) OR (thrombosis, intracranial[MeSH Terms])) OR (thrombus, brain[MeSH Terms])) OR (thrombus, cerebral[MeSH Terms])) OR (thrombus, intracranial[MeSH Terms]))) OR (((upper extremity deep vein thrombosis[MeSH Terms]) OR (central venous catheter thrombosis[MeSH Terms])) OR (upper extremity deep vein thrombosis, secondary[MeSH Terms]))) OR ((((((cavernous sinus thrombosis[MeSH Terms]) OR (cavernous sinus thromboses[MeSH Terms])) OR (sinus thromboses, cavernous[MeSH Terms])) OR (sinus thrombosis, cavernous[MeSH Terms])) OR (thromboses, cavernous sinus[MeSH Terms])) OR (thrombosis, cavernous sinus[MeSH Terms]))) OR (((sinus thrombosis, intracranial[MeSH Terms]) OR (cranial sinus thromboses[MeSH Terms])) OR (cranial sinus thrombosis[MeSH Terms]))) OR ((((((((((((((((((((((((((((((((((((((((((((((((((((((cerebral venous sinus thrombosis) OR (cerebrovascular sinus thrombosis)) OR (cerebrovascular venous sinus thrombosis)) OR (cranial venous sinus thrombosis)) OR (intracranial sinus thrombosis)) OR (intracranial venous sinus thrombosis)) OR (vena thrombosis)) OR (atherothrombosis)) OR (intravascular thrombosis)) OR (thrombo-occlusive)) OR (postoperative thrombosis)) OR (vein thromboembolism)) OR (deep vein blood clots)) OR (deep vein thrombus)) OR (lower extremity deep vein thrombosis)) OR (pulmonary thrombosis)) OR (lung thrombosis)) OR (lung thrombus)) OR (lung vessel thrombosis)) OR (pulmonary thrombus)) OR (pulmonary vascular thrombosis)) OR (pulmonary vessel thrombosis)) OR (pulmonary artery thrombosis)) OR (lung artery thrombosis)) OR (pulmonary arterial thrombosis)) OR (pulmonary artery thrombus)) OR (pulmonary vein thrombosis)) OR (pulmonary vein thrombus)) OR (cerebral sinus thrombosis)) OR (brain sinus thrombosis)) OR (brain venous sinus thrombosis)) OR (dura mater sinus thrombosis)) OR (dural venous sinus thrombosis)) OR (lung embolism)) OR (lung embolus)) OR (lung emboly)) OR (lung microembolism)) OR (lung microembolus)) OR (lung thromboembolism)) OR (pulmonary embolus)) OR (pulmonary microembolism)) OR (brain bleeding)) OR (brain microhemorrhage)) OR (cerebral microbleed)) OR (corpus callosum bleeding)) OR (corpus callosum hemorrhage)) OR (intracerebral bleeding)) OR (intracortical hemorrhage)) OR (intracranial bleeding)) OR (intracranial hemorrhage)) OR (major bleeding)) OR (periventricular hemorrhage)) OR (tumor bleeding)) OR (tumor hemorrhage))) OR (((((((((venous thromboembolism[MeSH Terms]) OR (pulmonary embolism[MeSH Terms])) OR (embolism, pulmonary[MeSH Terms])) OR (embolisms, pulmonary[MeSH Terms])) OR (pulmonary embolisms[MeSH Terms])) OR (pulmonary thromboembolism[MeSH Terms])) OR (pulmonary thromboembolisms[MeSH Terms])) OR (thromboembolism, pulmonary[MeSH Terms])) OR (thromboembolisms, pulmonary[MeSH Terms]))) OR (((((((((((((((((((((((((((((((((((((brain hemorrhage[MeSH Terms]) OR (intracranial hemorrhages[MeSH Terms])) OR (brain hemorrhage[MeSH Terms])) OR (brain hemorrhages[MeSH Terms])) OR (hemorrhage, brain[MeSH Terms])) OR (hemorrhage, intracranial[MeSH Terms])) OR (hemorrhage, posterior fossa[MeSH Terms])) OR (hemorrhages, brain[MeSH Terms])) OR (hemorrhages, intracranial[MeSH Terms])) OR (hemorrhages, posterior fossa[MeSH Terms])) OR (intracranial hemorrhage[MeSH Terms])) OR (posterior fossa hemorrhage[MeSH Terms])) OR (posterior fossa hemorrhages[MeSH Terms])) OR (brain hemorrhage, cerebral[MeSH Terms])) OR (brain hemorrhages, cerebral[MeSH Terms])) OR (cerebral brain hemorrhage[MeSH Terms])) OR (cerebral brain hemorrhages[MeSH Terms])) OR (cerebral hemorrhages[MeSH Terms])) OR (cerebral hemorrhages[MeSH Terms])) OR (cerebral parenchymal hemorrhage[MeSH Terms])) OR (cerebral parenchymal hemorrhages[MeSH Terms])) OR (cerebrum hemorrhage[MeSH Terms])) OR (cerebrum hemorrhages[MeSH Terms])) OR (hemorrhage, cerebral[MeSH Terms])) OR (hemorrhage, cerebral brain[MeSH Terms])) OR (hemorrhage, cerebral parenchymal[MeSH Terms])) OR (hemorrhage, cerebrum[MeSH Terms])) OR (hemorrhage, intracerebral[MeSH Terms])) OR (hemorrhages, cerebral[MeSH Terms])) OR (hemorrhages, cerebral brain[MeSH Terms])) OR (hemorrhages, cerebral parenchymal[MeSH Terms])) OR (hemorrhages, cerebrum[MeSH Terms])) OR (hemorrhages, intracerebral[MeSH Terms])) OR (intracerebral hemorrhage[MeSH Terms])) OR (intracerebral hemorrhages[MeSH Terms])) OR (parenchymal hemorrhage, cerebral[MeSH Terms])) OR (parenchymal hemorrhages, cerebral[MeSH Terms]))) OR (((((((((((((((((((((((((((((ganglionic hemorrhage) OR (basal ganglionic hemorrhage[MeSH Terms])) OR (ganglionic hemorrhage, basal[MeSH Terms])) OR (hematoma, basal ganglia[MeSH Terms])) OR (hemorrhage, basal ganglia[MeSH Terms])) OR (hemorrhage, basal ganglionic[MeSH Terms])) OR (intracerebral hemorrhage)) OR (cerebral intraventricular hemorrhage)) OR (cerebral intraventricular hemorrhages)) OR (cerebral intraventricular haemorrhage)) OR (cerebral intraventricular haemorrages)) OR (haemorrhage, cerebral intraventricular)) OR (hemorrhage, cerebral intraventricular)) OR (intraventricular haemorrhage, cerebral)) OR (intraventricular haemorrhages, cerebral)) OR (intraventricular hemorrhage, cerebral)) OR (posterior fossa hemorrhage)) OR (brain hemorrhage[MeSH Terms])) OR (brain hemorrhages[MeSH Terms])) OR (hemorrhage, brain[MeSH Terms])) OR (hemorrhage, intracranial[MeSH Terms])) OR (hemorrhage, posterior fossa[MeSH Terms])) OR (hemorrhages, brain[MeSH Terms])) OR (hemorrhages, intracranial[MeSH Terms])) OR (hemorrhages, posterior fossa[MeSH Terms])) OR (intracranial hemorrhage[MeSH Terms])) OR (posterior fossa hemorrhage[MeSH Terms])) OR (posterior hemorrhages[MeSH Terms])) OR (hemorragic shock)** | | |
| #3 | (incidence) OR (prevalence) OR (incidence[MeSH Terms]) OR (incidence studies[MeSH Terms]) OR (incidence study[MeSH Terms]) OR (incidences[MeSH Terms]) OR (studies, incidence[MeSH Terms]) OR (prevalence[MeSH Terms]) OR (prevalence studies[MeSH Terms]) OR (prevalence study[MeSH Terms]) OR (hazard ratio) OR (cox proportional hazards models[MeSH Terms]) OR (hazard model, proportional[MeSH Terms])) OR (hazard models, proportional[MeSH Terms]) OR (hazards model, proportional[MeSH Terms])) OR (odds ratio[MeSH Terms])) OR (odds ratios[MeSH Terms]) | | |
| #4 | #1 AND #2 AND #3 | | |
| #5 | #4 with filters: Full text, Case Reports, Observational Study, in the last 10 years, English, Portuguese, Spanish, Adult: 19+ years, Exclude preprints | | |
| Number of citations | 440 | | |
| Database | Cochrane Library | **Date** | 03/07 |
| #1 | (central nervous system neoplasms) OR (CNS neoplasm) OR (CNS neoplasms) OR (central nervous system neoplasm) OR (central nervous system neoplasms, primary) OR (central nervous system tumor) OR (central nervous system tumors) OR (neoplasm, CNS) OR (neoplasms, CNS) OR (neoplasms, central nervous system) OR (primary central nervous system neoplasm) OR (primary central nervous system neoplasms) OR (tumors central nervous system) OR (brain neoplasms) OR (brain cancer) OR (brain cancers) OR (brain malignant neoplasm) OR (brain malignant neoplasms) OR (brain neoplasm) OR (brain neoplasm, malignant) OR (brain neoplasm, primary) OR (brain neoplasms, malignant) OR (brain neoplasms, malignant, primary) OR (brain neoplasms, primary) OR (brain neoplasms, primary malignant) OR (brain tumor) OR (brain tumor, primary) OR (brain tumor, recurrent) OR (brain tumors) OR (brain tumors, primary) OR (brain tumors, recurrent) OR (cancer of brain) OR (cancer of the brain) OR (cancer, brain) OR (cancers, brain) OR (intracranial neoplasm) OR (intracranial neoplasms) OR (malignant brain neoplasm) OR (malignant brain neoplasms) OR (malignant neoplasm, brain) OR (malignant neoplasms, brain) OR (malignant primary brain neoplasms) OR (malignant primary brain tumors) OR (neoplasm, brain) OR (neoplasm, intracranial) OR (neoplasms, brain) OR (neoplasms, brain, malignant) OR (neoplasms, brain, primary) OR (neoplasms, intracranial) OR (primary brain neoplasm) OR (primary brain neoplasms) OR (primary brain tumor) OR (primary brain tumors) OR (primary malignant brain neoplasms) OR (primary malignant brain tumors) OR (recurrent brain tumor) OR (recurrent brain tumors) OR (tumor, brain) OR (tumors, brain) OR (cerebral ventricle neoplasm) OR (brain ventricular neoplasm) OR (brain ventricular neoplasms) OR (brain ventricular tumor) OR (brain ventricular tumors) OR (cerebral ventricle neoplasm) OR (cerebral ventricle tumor) OR (cerebral ventricle tumors) OR (cerebroventricular neoplasm) OR (cerebroventricular neoplasms) OR (intraventricular neoplasm) OR (intraventricular neoplasms) OR (neoplasm, brain ventricular) OR (neoplasm, cerebral ventricle) OR (neoplasm, cerebroventricular) OR (neoplasm, intraventricular) OR (neoplasms, brain ventricular) OR (neoplasms, cerebral ventricle) OR (neoplasms, cerebroventricular) OR (neoplasms, intraventricular) OR (neoplasms, ventricular, brain) OR (tumor, brain ventricular) OR (tumor, cerebral ventricle) OR (tumors, brain ventricular) OR (tumors, cerebral ventricle) OR (ventricle tumor, cerebral) OR (ventricle tumors, cerebral) OR (ventricular neoplasm, brain) OR (ventricular neoplasms, brain) OR (ventricular tumor, brain) OR (ventricular tumors, brain) OR (astroblastoma) OR (astroblastomas) OR (ependymoastrocytoma) OR (ependymoastrocytomas) OR (gliomatosis cerebri) OR (neoplasm, neuroepithelial) OR (neuroepithelial neoplasm) OR (neuroepithelial neoplasms) OR (neuroepithelial tumor) OR (neuroepithelial tumors) OR (polar spongioblastoma) OR (polar spongioblastomas) OR (spongioblastoma, polar) OR (spongioblastomas, polar) OR (tumor, neuroepithelial) OR (tumors, neuroepithelial) OR (neuroectodermal tumors, primitive) OR (cerebral primitive neuroectodermal tumor) OR (ependymoblastoma) OR (ependymoblastomas) OR (neoplasm, primitive neuroepithelial) OR (neoplasms, primitive neuroepithelial) OR (neuroectodermal tumor, primitive) OR (neuroepithelial neoplasm, primitive) OR (neuroepithelial neoplasm, primitive) OR (neuroepithelial neoplasms, primitive) OR (neuroepithelial tumor, primitive) OR (neuroepithelial tumors, primitive) OR (primitive neuroectodermal tumor) OR (primitive neuroectodermal tumors) OR (primitive neuroepithelial neoplasm) OR (primitive neuroepithelial neoplasms) OR (primitive neuroepithelial tumor) OR (primitive neuroepithelial tumors) OR (spongioblastoma) OR (spongioblastomas) OR (tumor, primitive neuroectodermal) OR (tumor, primitive neuroepithelial) OR (tumors, primitive neuroectodermal) OR (tumors, primitive neuroepithelial) OR (meningeal neoplasms) OR (cancer, meningeal) OR (cancers, meningeal) OR (intracranial meningeal neoplasm) OR (intracranial meningeal neoplasms) OR (leptomeningeal neoplasm) OR (leptomeningeal neoplasms) OR (meningeal cancer) OR (meningeal cancers) OR (meningeal neoplasm) OR (meningeal neoplasm, intracranial) OR (meningeal neoplasm, malignant) OR (meningeal neoplasm, spinal) OR (meningeal neoplasms, intracranial) OR (meningeal neoplasms, malignant) OR (meningeal neoplasms, spinal) OR (meningeal tumor) OR (meningeal tumors) OR (neoplasm, intracranial meningeal) OR (neoplasm, leptomeningeal) OR (neoplasm, malignant meningeal) OR (neoplasm, meningeal) OR (neoplasm, spinal meningeal) OR (neoplasms, intracranial meningeal) OR (neoplasms, leptomeningeal) OR (neoplasms, malignant meningeal) OR (neoplasms, meningeal) OR (neoplasms, spinal meningeal) OR (spinal meningeal neoplasm) OR (spinal meningeal neoplasms) OR (tumor, meningeal) OR (tumors, meningeal) OR (glioma) OR (glial cell tumor) OR (glial cell tumors) OR (glioma, malignant) OR (glioma, mixed) OR (gliomas) OR (gliomas, malignant) OR (gliomas, mixed) OR (malignant glioma) OR (malignant gliomas) OR (mixed glioma) OR (mixed gliomas) OR (tumor, glial cell) OR (tumors, glial cell) OR (ganglioglioma) OR (ganglioglioma, intracranial) OR (ganglioglioma, malignant) OR (gangliogliomas) OR (gangliogliomas, intracranial) OR (gangliogliomas, malignant) OR (intracranial ganglioglioma) OR (intracranial gangliogliomas) OR (malignant ganglioglioma) OR (malignant gangliogliomas) OR (astrocytoma) OR (anaplastic astrocytoma) OR (anaplastic astrocytomas) OR (astrocytic glioma) OR (astrocytic gliomas) OR (astrocytoma, anaplastic) OR (astrocytoma, cerebral) OR (astrocytoma, fibrillary) OR (astrocytoma, gemistocytic) OR (astrocytoma, intracranial) OR (astrocytoma, pilocytic) OR (astrocytoma, protoplasmic) OR (astrocytoma, subependymal) OR (astrocytomas) OR (astrocytomas, anaplastic) OR (astrocytomas, cerebral) OR (astrocytomas, fibrillary) OR (astrocytomas, gemistocytic) OR (astrocytomas, intracranial) OR (astrocytomas, pilocytic) OR (astrocytomas, protoplasmic) OR (astroglioma) OR (astrogliomas) OR (cerebral astrocytoma) OR (cerebral astrocytomas) OR (fibrillary astrocytoma) OR (fibrillary astrocytomas) OR (gemistocytic astrocytoma) OR (gemistocytic astrocytomas) OR (glioma, astrocytic) OR (gliomas, astrocytic) OR (intracranial astrocytoma) OR (intracranial astrocytomas) OR (mixed oligoastrocytoma) OR (mixed oligoastrocytomas) OR (oligoastrocytoma, mixed) OR (oligoastrocytomas, mixed) OR (pilocytic astrocytoma) OR (pilocytic astrocytomas) OR (pleomorphic xanthoastrocytomas) OR (protoplasmic astrocytoma) OR (protoplasmic astrocytomas) OR (subependymal giant cell astrocytoma) OR (glioblastoma) OR (giant cell glioblastoma) OR (giant cell glioblastomas) OR (glioblastoma, giant cell) OR (glioblastoma multiforme) OR (glioblastomas) OR (glioblastomas, giant cell) OR (ependymoma) OR (anaplastic ependymoma) OR (anaplastic ependymomas) OR (cellular ependymoma) OR (clear cell ependymoma) OR (ependymoma, anaplastic) OR (ependymoma, myxopapillary) OR (ependymoma, papillary) OR (ependymomas) OR (ependymomas, anaplastic) OR (ependymomas, myxopapillary) OR (ependymomas, papillary) OR (myxopapillary ependymoma) OR (myxopapillary ependymomas) OR (papillary ependymoma) OR (papillary ependymomas) OR (oligodendroglioma) OR (adult oligodendroglioma) OR (adult oligodendrogliomas) OR (anaplastic oligodendroglioma) OR (anaplastic oligodendrogliomas) OR (mixed oligodendroglioma astrocytoma) OR (mixed oligodendroglioma ependymoma) OR (oligodendroblastoma) OR (oligodendroblastomas) OR (oligodendroglioma, adult) OR (oligodendroglioma, anaplastic) OR (oligodendroglioma, well differentiated) OR (oligodendrogliomas) OR (oligodendrogliomas, adult) OR (oligodendrogliomas, anaplastic) OR (well diferentiated oligodendroglioma) OR (glioma, subependymal) OR (adult subependymal astrocytoma) OR (adult subependymal astrocytomas) OR (astrocytoma, adult subependymal) OR (astrocytoma, subependymal) OR (astrocytomas, subependymal) OR (astrocytomas, adult subependymal) OR (gliomas, subependymal) OR (glioses, subependymal) OR (gliosis, subependymal) OR (subependymal astrocytoma) OR (subependymal astrocytoma, adult) OR (subependymal astrocytomas) OR (subependymal astrocytomas, adult) OR (subependymal glioma) OR (subependymal gliomas) OR (subependymal glioses) OR (subependymal gliosis) OR (subependymoma) OR (subependymomas) OR (malignant meningioma) OR (malignant meningiomas) | | |
| #2 | (cerebral hemorrhage) OR (cerebrum hemorrhage) OR (cerebrum hemorrhages) OR (cerebral parenchymal hemorrhage) OR (brain hemorrhage, cerebral) OR (brain hemorrhages, cerebral) OR (cerebral brain hemorrhage) OR (cerebral brain hemorrhages) OR (cerebral hemorrhages) OR (cerebral parenchymal hemorrhage) OR (cerebral parenchymal hemorrhages) OR (cerebrum hemorrhage) OR (cerebrum hemorrhages) OR (hemorrhage, cerebral) OR (hemorrhage, cerebral brain) OR (hemorrhage, cerebral parenchymal) OR (hemorrhage, cerebrum) OR (hemorrhage, intracerebral) OR (hemorrhages, cerebral) OR (hemorrhages, cerebral brain) OR (hemorrhages, cerebral parenchymal) OR (hemorrhages, cerebrum) OR (hemorrhages, intracerebral) OR (intracerebral hemorrhage) OR (intracerebral hemorrhages) OR (parenchymal hemorrhage, cerebral) OR (parenchymal hemorrhages, cerebral) OR (thrombosis) OR (venous thrombosis) OR (thrombosis, deep vein) OR (thrombosis, deep venous) OR (thrombosis, venous) OR (vein thromboses, deep) OR (vein thrombosis, deep) OR (venous thromboses) OR (venous thromboses, deep) OR (venous thrombosis, deep) OR (intracranial thrombosis) OR (brain thromboses) OR (brain thrombosis) OR (brain thrombus) OR (cerebral thromboses) OR (cerebral thrombosis) OR (cerebral thrombus) OR (intracranial thromboses) OR (intracranial thrombus) OR (thromboses, brain) OR (thromboses, cerebral) OR (thromboses, cerebral) OR (thromboses, intracranial) OR (thrombosis, brain) OR (thrombosis, cerebral) OR (thrombosis, intracranial) OR (thrombus, brain) OR (thrombus, cerebral) OR (thrombus, intracranial) OR (upper extremity deep vein thrombosis) OR (central venous catheter thrombosis) OR (upper extremity deep vein thrombosis, secondary) OR (cavernous sinus thrombosis) OR (cavernous sinus thromboses) OR (sinus thromboses, cavernous) OR (sinus thrombosis, cavernous) OR (thromboses, cavernous sinus) OR (thrombosis, cavernous sinus) OR (sinus thrombosis, intracranial) OR (cranial sinus thromboses) OR (cranial sinus thrombosis) OR (cerebral venous sinus thrombosis) OR (venous thromboembolism) OR (pulmonary embolism) OR (embolism, pulmonary) OR (embolisms, pulmonary) OR (pulmonary embolisms) OR (pulmonary thromboembolism) OR (pulmonary thromboembolisms) OR (thromboembolism, pulmonary) OR (thromboembolisms, pulmonary) OR (brain hemorrhage) OR (intracranial hemorrhages) OR (brain hemorrhage) OR (brain hemorrhages) OR (hemorrhage, brain) OR (hemorrhage, intracranial) OR (hemorrhage, posterior fossa) OR (hemorrhages, brain) OR (hemorrhages, intracranial) OR (hemorrhages, posterior fossa) OR (intracranial hemorrhage) OR (posterior fossa hemorrhage) OR (posterior fossa hemorrhages) OR (brain hemorrhage, cerebral) OR (brain hemorrhages, cerebral) OR (cerebral brain hemorrhage) OR (cerebral brain hemorrhages) OR (cerebral hemorrhages) OR (cerebral hemorrhages) OR (cerebral parenchymal hemorrhage) OR (cerebral parenchymal hemorrhages) OR (cerebrum hemorrhage) OR (cerebrum hemorrhages) OR (hemorrhage, cerebral) OR (hemorrhage, cerebral brain) OR (hemorrhage, cerebral parenchymal) OR (hemorrhage, cerebrum) OR (hemorrhage, intracerebral) OR (hemorrhages, cerebral) OR (hemorrhages, cerebral brain) OR (hemorrhages, cerebral parenchymal) OR (hemorrhages, cerebrum) OR (hemorrhages, intracerebral) OR (intracerebral hemorrhage) OR (intracerebral hemorrhages) OR (parenchymal hemorrhage, cerebral) OR (parenchymal hemorrhages, cerebral) OR (ganglionic hemorrhage) OR (basal ganglionic hemorrhage) OR (ganglionic hemorrhage, basal) OR (hematoma, basal ganglia) OR (hemorrhage, basal ganglia) OR (hemorrhage, basal ganglionic) OR (intracerebral hemorrhage) OR (cerebral intraventricular hemorrhage) OR (cerebral intraventricular hemorrhages) OR (cerebral intraventricular haemorrhage) OR (cerebral intraventricular haemorrages) OR (haemorrhage, cerebral intraventricular) OR (hemorrhage, cerebral intraventricular) OR (intraventricular haemorrhage, cerebral) OR (intraventricular haemorrhages, cerebral) OR (intraventricular hemorrhage, cerebral) OR (posterior fossa hemorrhage) OR (brain hemorrhage) OR (brain hemorrhages) OR (hemorrhage, brain) OR (hemorrhage, intracranial) OR (hemorrhage, posterior fossa) OR (hemorrhages, brain) OR (hemorrhages, intracranial) OR (hemorrhages, posterior fossa) OR (intracranial hemorrhage) OR (posterior fossa hemorrhage) OR (posterior hemorrhages) OR (hemorragic shock) | | |
| #3 | MeSH descriptor: [Incidence] explode all trees | | |
| #4 | MeSH descriptor: [Prevalence] explode all trees | | |
| #5 | MeSH descriptor: [Cohort Studies] explode all trees | | |
| #6 | MeSH descriptor: [Observational Study] explode all trees | | |
| #7 | (#1 AND #2) AND (#3 OR #4 OR #5 OR #6) with Publication Year from 2013 to 2023, with Cochrane Library publication date Between Jan 2013 and Jul 2023, in Trials (Word variations have been searched) | | |
| Number of citations | 88 | | |
| Database | BVS | **Date** | 03/07 |
| #1 | ((central nervous system neoplasm*) OR (central nervous system neoplasm*, primary) OR (central nervous system tumor*) OR (neoplasm*, central nervous system) OR (primary central nervous system neoplasm*) OR (tumor* central nervous system) OR (brain neoplasm*) OR (brain cancer*) OR (brain malignant neoplasm*) OR (brain neoplasm*, primary) OR (brain neoplasm*, malignant) OR (brain neoplasm*, malignant, primary) OR (brain tumor*, primary) OR (brain tumor*, recurrent) OR (cancer of brain) OR (cancer of the brain) OR (cancer*, brain) OR (intracranial neoplasm*) OR (malignant brain neoplasm*) OR (malignant primary brain neoplasm*) OR (malignant primary brain tumor*) (neoplasm*, brain, malignant) OR (neoplasm*, brain, primary) OR (neoplasm*, intracranial) OR (primary brain neoplasm*) OR (primary brain tumor*) OR (primary malignant brain neoplasm*) OR (recurrent brain tumor*) OR (cerebral ventricle neoplasm*) OR (brain ventricular neoplasm*) OR (brain ventricular tumor*) OR (cerebral ventricle tumor*) OR (cerebroventricular neoplasm*) OR (intraventricular neoplasm*) OR (astroblastoma*) OR (ependymoastrocytoma*) OR (gliomatosis cerebri) OR (neoplasm*, neuroepithelial) OR (neuroepithelial neoplasm*) OR (neuroepithelial tumor*) OR (polar spongioblastoma*) OR (spongioblastoma*, polar) OR (neuroectodermal tumor*, primitive) OR (cerebral primitive neuroectodermal tumor*) OR (ependymoblastoma*) OR (neoplasm*, primitive neuroepithelial) OR (neuroectodermal tumor*, primitive) OR (neuroepithelial neoplasm*, primitive) OR (neuroepithelial tumor*, primitive) OR (primitive neuroectodermal tumor*) OR (primitive neuroepithelial neoplasm*) OR (primitive neuroepithelial tumor*) OR (spongioblastoma*) OR (tumor*, primitive neuroectodermal) OR (tumor*, primitive neuroepithelial) OR (meningeal neoplasm*) OR (cancer*, meningeal) OR (intracranial meningeal neoplasm*) OR (leptomeningeal neoplasm*) OR (meningeal cancer*) OR (meningeal neoplasm*) OR (meningeal neoplasm*, intracranial) OR (meningeal neoplasm*, malignant) OR (meningeal neoplasm*, spinal) OR (meningeal tumor*) OR (neoplasm*, intracranial meningeal) OR (neoplasm*, leptomeningeal) OR (neoplasm*, malignant meningeal) OR (neoplasm*, meningeal) OR (neoplasm*, spinal meningeal) OR (spinal meningeal neoplasm*) OR (tumor*, meningeal) OR (glial cell tumor*) OR (glioma*, malignant) OR (glioma*, mixed) OR (glioma*) OR (malignant glioma*) OR (mixed glioma*) OR (tumor*, glial cell) OR (ganglioglioma*) OR (ganglioglioma*, intracranial) OR (ganglioglioma*, malignant) OR (intracranial ganglioglioma*) OR (malignant ganglioglioma*) OR (astrocytoma*) OR (anaplastic astrocytoma*) OR (astrocytic glioma*) OR (astrocytoma*, anaplastic) OR (astrocytoma*, cerebral) OR (astrocytoma*, fibrillary) OR (astrocytoma*, gemistocytic) OR (astrocytoma*, intracranial) OR (astrocytoma*, pilocytic) OR (astrocytoma*, protoplasmic) OR (astrocytoma*, subependymal) OR (astroglioma*) OR (cerebral astrocytoma*) OR (fibrillary astrocytoma*) OR (gemistocytic astrocytoma*) OR (glioma*, astrocytic) OR (intracranial astrocytoma*) OR (mixed oligoastrocytoma*) OR (oligoastrocytoma*, mixed) OR (pilocytic astrocytoma*) OR (pleomorphic xanthoastrocytomas) OR (protoplasmic astrocytoma*) OR (subependymal giant cell astrocytoma*) OR (glioblastoma*) OR (giant cell glioblastoma*) OR (glioblastoma, giant cell) OR (glioblastoma multiforme) OR (glioblastoma*, giant cell) OR (anaplastic ependymoma*) OR (cellular ependymoma) OR (clear cell ependymoma) OR (ependymoma*) OR (ependymoma*, anaplastic) OR (ependymoma*, myxopapillary) OR (ependymoma*, papillary) OR (myxopapillary ependymoma*) OR (papillary ependymoma*) OR (oligodendroglioma*) OR (adult oligodendroglioma*) OR (anaplastic oligodendroglioma*) OR (mixed oligodendroglioma astrocytoma) OR (mixed oligodendroglioma ependymoma) OR (oligodendroblastoma*) OR (oligodendroglioma*, adult) OR (oligodendroglioma*, anaplastic) OR (well diferentiated oligodendroglioma) OR (glioma, subependymal) OR (adult subependymal astrocytoma*) OR (astrocytoma*, adult subependymal) OR (astrocytoma*, subependymal) OR (glio*, subependymal) OR (subependymal astrocytoma*) OR (subependymal astrocytomas, adult) OR (subependymal glioma*) OR (subependymal glios*) OR (subependymoma*) OR (malignant meningioma*)) | | |
| #2 | ((cerebr* hemorrhage*) OR (cerebr* parenchymal hemorrhage*) OR (cerebr* brain hemorrhage*) OR (intracerebral hemorrhage*) OR (thromb*) OR (venous thromb*) OR (thromb*, deep vein) OR (vein thromb*, deep) OR (venous thromb*) OR (intracranial thromb*) OR (brain thromb*) OR (cerebr* thromb*) OR (intracranial thromb*) OR (thromb*, brain) OR (thromb*, cerebr*) OR (thromb*, intracranial) OR (upper extremity deep vein thromb*) OR (central venous catheter thromb*) OR (upper extremity deep vein thromb*, secondary) OR (cavernous sinus thromb*) OR (sinus thromb*, intracranial) OR (cranial sinus thromb*) OR (cerebral venous sinus thromb*) OR (cerebrovascular sinus thromb*) OR (intracranial venous sinus thromb*) OR (venous thromboembolism) OR (pulmonary embolism*) OR (pulmonary thromboembolism*) OR (brain hemorrhage*) OR (intracranial hemorrhage*) OR (posterior fossa hemorrhage*) OR (brain hemorrhage*, cerebral) OR (cerebral hemorrhage*) OR (cerebral parenchymal hemorrhage*) OR (cerebr* hemorrhage*) OR (ganglionic hemorrhage) OR (basal ganglionic hemorrhage) OR (ganglionic hemorrhage, basal) OR (hematoma, basal ganglia) OR (hemorrhage, basal ganglia) OR (hemorrhage, basal ganglionic) OR (intracerebral hemorrhage) OR (cerebral intraventricular hemorrhage*) OR (hemorrhage, cerebral intraventricular) OR (intraventricular hemorrhage, cerebral) OR (posterior fossa hemorrhage) OR (brain hemorrhage*) OR (hemorrhage*, brain) OR (hemorrhage*, intracranial) OR (hemorrhage*, posterior fossa) OR (intracranial hemorrhage) OR (posterior fossa hemorrhage) OR (hemorragic shock)) | | |
| #3 | ((incidence) OR (prevalence) OR (observational stud*) OR (cohort stud*) OR (hazard ratio) OR (odds ratio)) | | |
| #4 | #1 AND #2 AND #3 | | |
| #5 | #4 AND (fulltext:("1") AND type_of_study:("observational_studies" OR "incidence_studies" OR "prevalence_studies") AND la:("en" OR "es" OR "pt")) AND (year_cluster:[2013 TO 2023]) | | |
| Number of citations | 1125 | | |
