# Supplementary material for: Incidence of venous thromboembolism and bleeding in patients with malignant central nervous system neoplasm: Systematic review and meta-analysis
Source: PLoS One. 2024 Jun 20;19(6):e0304682. doi: 10.1371/journal.pone.0304682 (PMC11189257; doi:10.1371/journal.pone.0304682)
Supplement: S2 Table — (DOCX) [file pone.0304682.s007.docx]

S2 Table. Characteristics of excluded studies (*ordered by study ID*).

| Study | Reason for exclusion |
| --- | --- |
| Smith et al., 2014 (doi: 10.1016/j.jocn.2014.10.003) | Duplicate |
| Booth and Pilz, 2022 (doi: 10.1177%2F00185787211046865) | Not retrieved |
| Zeiner et al., 2022 (doi: 10.1212/WNL.0000000000200254) | Not retrieved |
| Rusconi et al., 2022 (doi: 10.1182/blood.2022015560) | Not retrieved |
| Zhang et al., 2021 (doi: 10.1097/PAS.0000000000001762) | Not retrieved |
| Lapébie et al., 2023 (doi: 10.1016/j.jtha.2023.04.010) | Not retrieved |
| Merenzon et al., 2023 (doi: 10.1227/ons.0000000000000553) | Not retrieved |
| Mahajan et al., 2022 (doi: 10.1055/a-1742-0177) | Not retrieved |
| Edupuganti et al., 2021 (doi: 10.1016/j.amjmed.2021.11.006) | Not retrieved |
| Taguchi et al., 2022 (doi: 10.1016/j.wneu.2021.10.161) | Not retrieved |
| Yamaura et al., 2021 (doi: 10.1016/j.thromres.2021.08.016) | Not retrieved |
| Squizzato et al., 2019 (doi: 10.1055/s-0039-1697661) | Not retrieved |
| Giustozzi et al., 2020 (doi: 10.1055/s-0040-1709527) | Not retrieved |
| Kobayashi et al., 2018 (doi: 10.1097/BRS.0000000000002496) | Not retrieved |
| Geraldes et al., 2017 (https://pubmed.ncbi.nlm.nih.gov/28650062/) | Not retrieved |
| Honorato-Cia et al., 2015 (doi: 10.1097/ANA.0000000000000172) | Not retrieved |
| Dasenbrock et al., 2017 (doi: 10.1093/neuros/nyx089) | Not retrieved |
| Faraji et al., 2020 (doi:  10.1093/ons/opaa029) | Not retrieved |
| Chai-Adisaksopha et al., 2017 (doi: 10.1160/TH16-09-0680) | Not retrieved |
| Ferroni et al., 2016 (doi: 10.1160/TH15-03-0259) | Not retrieved |
| Dickinson et al., 2014 (doi: 10.3171/2014.8.JNS1498) | Not retrieved |
| Borde et al., 2017 (doi: 10.4103/neuroindia.NI_1237_15) | Not retrieved |
| Botros et al., 2022 (doi: 10.1227/neu.0000000000002063) | Not retrieved |
| Ma et al., 2023 (doi: 10.1016/j.jtha.2023.01.031) | Not retrieved |
| Shabo et al., 2023 (doi: 10.1227/neu.0000000000002340) | Outcome |
| Fang et al., 2022 (doi: 10.1155/2022/4516537) | Outcome |
| Li, Deng, Su, 2022 (doi: 10.1016/j.wneu.2022.06.089) | Outcome |
| Kikuno et al., 2021 (doi: 10.1161/JAHA.120.021375) | Outcome |
| Armahizer et al., 2021 (doi: 10.1016/j.wneu.2020.11.107) | Outcome |
| Theriault et al., 2020 (doi: 10.3171/2020.7.FOCUS20324) | Outcome |
| Schneider et al., 2020 (doi: 10.1007/s11060-020-03629-y) | Outcome |
| Topkan et al., 2020 (doi: 10.1155/2020/4392189) | Outcome |
| Chen et al., 2020 (doi: 10.1042/BSR20201449) | Outcome |
| Zhang et al., 2019 (doi: 10.1016/j.jocn.2019.07.067) | Outcome |
| Yerram et al., 2020 (doi: 10.1007/s11060-019-03266-0) | Outcome |
| Chen et al., 2016 (doi: 10.1016/j.jns.2016.10.022) | Outcome |
| Bertoli et al., 2018 (doi: 10.1080/01635581.2018.1446089) | Outcome |
| Perez et al., 2020 (doi: 10.1016/j.jcrc.2020.08.013) | Outcome |
| Brenner et al., 2019 (doi: 10.1159/000500926) | Outcome |
| Maslink et al., 2019 (doi: 10.1016/j.wneu.2019.06.093) | Outcome |
| Zipser et al., 2019 (doi: 10.1007/s00701-019-03927-z) | Outcome |
| Sgreccia et al., 2019 (https://jnis.bmj.com/content/11/12/1197) | Outcome |
| Petr et al., 2016 (doi: 10.1016/j.radonc.2015.12.017) | Outcome |
| Chang et al., 2020 (doi: 10.1016/j.clml.2020.08.020) | Outcome |
| Kaji et al., 2021 (doi: 10.1111/bjh.17747) | Outcome |
| Maas et al., 2019 (doi: 10.1016/j.wneu.2019.03.053) | Outcome |
| Morrison et al., 2019 (doi: 10.1002/jmri.26651) | Outcome |
| Collignon et al., 2019 (doi: 10.1007/s00277-018-3564-6) | Outcome |
| Bao et al., 2018 (doi: 10.1016/j.wneu.2018.07.252) | Outcome |
| Sahin et al., 2023 (doi: 10.1016/j.clml.2022.09.006) | Outcome |
| Franceschi et al., 2016 (doi: 10.1007/s11060-016-2093-1) | Outcome |
| Isono et al., 2021 (doi: 10.1002/prp2.883) | Outcome |
| Venkatraghavan et al., 2016 (doi: 10.1007/s12630-016-0717-8) | Outcome |
| Heath et al., 2018 (doi: 10.21873/anticanres.12466) | Outcome |
| Bacqué et al., 2018 (doi: 10.1016/j.transproceed.2017.11.074) | Outcome |
| Campian et al., 2017 (doi: 10.1007/s11060-017-2580-z) | Outcome |
| Fujio et al., 2017 (doi: 10.2176/nmc.oa.2017-0079) | Outcome |
| Sun et al., 2015 (doi: 10.1016/j.wneu.2015.04.057) | Outcome |
| Medhi et al., 2016 (doi: 10.1007/s00330-015-3808-y) | Outcome |
| Ali et al., 2020 (doi: 10.12659/AJCR.917694) | Outcome |
| Mason et al., 2014 (doi: 10.1136/bcr-2014-208117) | Outcome |
| Lefranc et al., 2015 (doi: 10.3171/2014.9.JNS14107) | Outcome |
| Martínez-Martínez, Bosch, 2014 (doi: 10.1016/j.rx.2013.02.006) | Outcome |
| Zhang et al., 2018 (doi: 10.1080/02688697.2017.1416059) | Outcome |
| http://kjfm.or.kr/journal/view.php?doi=10.4082/kjfm.2017.38.4.220 | Outcome |
| van Linde et al., 2016 (doi: 10.1007/s11060-016-2178-x) | Outcome |
| Russo et al., 2017 (doi: 10.1007/s00381-017-3434-x) | Outcome |
| Subeikshanan et al., 2016 (doi: 10.4103/0022-3859.180551) | Outcome |
| O’Leary et al., 2016 (doi: 10.1007/s11060-015-2033-5) | Outcome |
| Ko et al., 2020 (doi: 10.1186/s40478-020-00991-w) | Outcome |
| Selvik et al., 2015 (doi: 10.1159/000440730) | Outcome |
| Blumenthal et al., 2015 (doi: 10.1007/s11060-015-1796-z) | Outcome |
| Qureshi et al., 2015 (doi: 10.1159/000375154) | Outcome |
| Mikhail et al., 2015 (doi: 10.1007/s12032-015-0622-y) | Outcome |
| Tinchon et al., 2015 (doi: 10.1007/s00415-014-7552-z) | Outcome |
| Ackerl et al. (doi: 10.5414/NP300761) | Outcome |
| Boström et al., 2013 (doi:10.1007/s00066-013-0505-0) | Outcome |
| Hamasaki, Yamada, Kuratsu, 2013 (doi: 10.1016/j.clineuro.2013.08.016) | Outcome |
| Hawasli et al., 2013 (doi: 10.1227/NEU.0000000000000144) | Outcome |
| Roelcke et al., 2013 (doi: 10.1007/s11060-013-1247-7) | Outcome |
| Babu et al., 2013 (doi: 10.1016/j.jocn.2012.12.011) | Outcome |
| Gupta et al., 2013 (doi: 10.1016/j.clineuro.2013.05.015) | Outcome |
| Shonka et al., 2013 (doi: 10.1007/s11523-013-0254-0) | Outcome |
| Kreisl et al., 2013 (doi: 10.1007/s11060-012-0988-z) | Outcome |
| Nakaji et al., 2022 (doi: 10.1093/neuonc/noac133) | Outcome |
| Smith et al., 2022 | Outcome |
| Duan et al., 2023 (doi: 10.1186/s12885-023-10889-0) | Outcome |
| Bosio et al., 2023 (doi: 10.1016/j.clon.2023.01.012) | Outcome |
| Ghodrati et al., 2023 (doi: 10.1007/s11060-023-04259-w) | Outcome |
| Wang et al., 2022 (doi: 10.1007/s11060-022-04162-w) | Outcome |
| Bhattacharjee et al., 2022 (doi: 10.1007/s00234-022-02946-5) | Outcome |
| Sander et al., 2021 (doi: 10.1016/j.wneu.2021.01.123) | Outcome |
| Woo et al., 2020 (doi: 10.1016/j.wneu.2020.09.027) | Outcome |
| Meling et al., 2019 (doi: 10.1016/j.wneu.2019.01.042) | Outcome |
| Huang and Lieu, 2020 (doi: 10.1097/MD.0000000000019226) | Outcome |
| Gessler et al., 2019 (doi: 10.1093/neuros/nyy272) | Outcome |
| Gutierrez et al., 2019 (doi: 10.1177/1753944719860676) | Outcome |
| Rodríguez-Florido et al., 2019 (doi: 10.24875/GMM.M20000329) | Outcome |
| Rinaldo et al., 2017 (doi: 10.3171/2017.6.JNS17859) | Outcome |
| Mantovani et al., 2014 (doi: 10.1016/j.wneu.2013.06.024) | Outcome |
| Kim et al., 2017 (doi: 10.1097/MD.0000000000006594) | Outcome |
| Ditzel Filho et al., 2014 (doi: 10.1016/j.wneu.2012.11.051) | Outcome |
| Lonjaret et al., 2017 (doi: 10.1016/j.accpm.2016.06.012) | Outcome |
| Di Vito et al., 2016 (doi: 10.1080/09537104.2016.1247208) | Outcome |
| Oh et al., 2014 (doi: 10.1016/j.clineuro.2013.12.017) | Outcome |
| Koutourousiou et al., 2014 (doi: 10.3171/2014.2.JNS13767) | Outcome |
| Lecler et al., 2018 (doi: 10.1007/s00330-018-5356-8) | Outcome |
| Cinotti et al., 2018 (doi: 10.1097/ALN.0000000000002426) | Outcome |
| Rodríguez-Florido et al., 2018 (doi: 10.24875/GMM.19004832) | Outcome |
| Luo et al., 2022 (doi: 10.1097/MD.0000000000032259) | Outcome |
| Marini et al., 2020 (doi: 10.1016/j.clineuro.2020.106162) | Outcome |
| Saad et al., 2021 (doi: 10.1093/neuros/nyab341) | Outcome |
| Khatri et al., 2021 (doi: 10.4103/aer.aer_136_21) | Outcome |
| Ryu et al., 2019 (doi: 10.1371/journal.pone.0215280) | Outcome |
| Ho et al., 2015 (doi: 10.1016/j.thromres.2015.02.002) | Outcome |
| Simonetti et al., 2020 (doi: 10.1007/s00520-019-05128-x) | Outcome |
| Elkady, Soliman, Ali, 2020 (doi: 10.1016/j.wneu.2020.01.202) | Outcome |
| Englisch et al., 2022 (doi: 10.1182/bloodadvances.2021006283) | Outcome (events were not presented according to cancer site/type) |
| Gheewala et al., 2021 (doi: 10.1016/j.jgo.2021.12.016) | Outcome (events were not presented according to cancer site/type) |
| Davies et al., 2021 (doi: 10.1111/imj.15353) | Outcome (events were not presented according to cancer site/type) |
| Kakkar et al., 2020 (doi: 10.1634/theoncologist.2019-0676) | Outcome (events were not presented according to cancer site/type) |
| Navi et al., 2018 (doi: 10.1212/WNL.0000000000005636) | Outcome (events were not presented according to cancer site/type) |
| Grazioli et al., 2018 (doi: 10.1016/j.thromres.2018.03.011) | Outcome (events were not presented according to cancer site/type) |
| Zuurbier et al., 2017 (doi: 10.1161/STROKEAHA.117.019483) | Outcome (events were not presented according to cancer site/type) |
| Dasenbrock et al., 2016 (doi: 10.3171/2016.2.JNS152345) | Outcome (events were not presented according to cancer site/type) |
| Khan et al., 2022 (doi: 10.1371/journal.pmed.1004012) | Outcome (events were not presented according to cancer site/type) |
| Chee et al., 2014 (doi: 10.1182/blood-2014-01-549733) | Outcome (events were not presented according to cancer site/type) |
| Nazari et al., 2018 (doi: 10.1111/jth.14129) | Outcome (events were not presented according to cancer site/type) |
| Ambulkar et al., 2023 (doi: 10.25259/JNRP_26_2022) | Outcome (events were not presented according to cancer site/type) |
| Krishnatry et al., 2021 (doi: 10.1093/jjco/hyab006) | Pediatric population |
| Michaiel et al., 2020 (doi: 10.1007/s11060-020-03486-9) | Pediatric population |
| Hu et al., 2023 (doi: 10.1097/MD.0000000000033116) | Population |
| Panic et al., 2022 (doi: 10.3390/idr14030045) | Population |
| Cohen et al., 2022 (doi: 10.1055/s-0042-1743470) | Population |
| Tomio et al., 2021 (doi: 10.3171/2020.12.JNS204010) | Population |
| Ungar et al., 2022 (doi: 10.1016/j.wneu.2021.07.063) | Population |
| Ganesh et al., 2021 (doi: 10.1001/jamanetworkopen.2021.32376) | Population |
| Rush and Rush, 2021 (doi: 10.1097/IAE.0000000000003276) | Population |
| Lahti et al., 2021 (doi: 10.1111/ene.14988) | Population |
| Lahti et al., 2021 (doi: 10.1016/j.eplepsyres.2021.106586) | Population |
| Maximilian et al., 2021 (doi: 10.1016/j.jvs.2021.01.058) | Population |
| Keogh et al., 2021 (doi: 10.1186%2Fs12885-021-07809-5) | Population |
| QiMin et al., 2020 (doi: 10.1186%2Fs13019-020-01364-z) | Population |
| Rozman et al., 2020 (doi: 10.1016/j.wneu.2020.07.207) | Population |
| de Souza et al., 2020 (doi: 10.1016/j.clineuro.2020.106128) | Population |
| Plestch-Borba et al., 2020 (doi: 10.1212/WNL.0000000000009391) | Population |
| https://www.jstage.jst.go.jp/article/circj/84/5/84_CJ-20-0241/_article | Population |
| Levraut et al., 2020 (doi: 10.1007/s11239-020-02100-z) | Population |
| Ehlers et al., 2020 (doi: 10.1016/j.clineuro.2020.105716) | Population |
| Kim et al., 2020 (doi: 10.3904/kjim.2017.415) | Population |
| Scordo et al., 2021 (doi: 10.1001/jamaoncol.2021.1074) | Population |
| Xie et al., 2014 (doi: 10.1016/j.jocn.2013.08.018) | Population |
| Delgrange et al., 2013 (doi: 10.1530/EJE-13-0503) | Population |
| Nawabi et al., 2020 (doi: 10.1007/s00062-019-00774-4) | Population |
| Reisinger et al., 2020 (doi: 10.1093/neuros/nyz148) | Population |
| Silvis et al., 2020 (doi: 10.1111/ene.14148) | Population |
| Zhong et al., 2020 (doi: 10.1016/j.wneu.2019.08.245) | Population |
| Deboeuf et al., 2023 (doi: 10.1227/neu.0000000000002294) | Population |
| Cheng et al., 2020 (doi: 10.1097/MD.0000000000018572) | Population |
| Xian et al., 2019 (doi: 10.1097/MD.0000000000015134) | Population |
| Burkhardt et al., 2019 (doi: 10.1055/s-0039-1685194) | Population |
| Feng et al., 2019 (doi: 10.1634/theoncologist.2018-0789) | Population |
| Kabrhel et al., 2014 (doi: 10.1136/thoraxjnl-2013-204762) | Population |
| Mathur, 2020 (doi: 10.1055/s-0040-1713383) | Population |
| Ryu, Bang, Lee, 2017 (doi: 10.1186/s12885-017-3588-7) | Population |
| Roach et al., 2019 (doi: 10.1016/j.wneu.2019.01.106) | Population |
| Ma et al., 2019 (doi: 10.3171/2018.8.JNS181580) | Population |
| Labberton et al., 2019 (doi: 10.1002/brb3.1175) | Population |
| Hu et al., 2019 (doi: 10.1016/j.amjcard.2018.11.04) | Population |
| Qi et al., 2018 (doi: 10.12659/MSM.911645) | Population |
| Hanak et al., 2016 (doi: 10.1136/neurintsurg-2015-011980) | Population |
| Price et al., 2018 (doi: 10.1212/WNL.0000000000004856) | Population |
| Corraini et al., 2018 (doi: 10.1111/jth.13908) | Population |
| Jin et al., 2018 (doi: 10.1161/STROKEAHA.117.019189) | Population |
| Quick-Weller et al., 2018 (doi: 10.1080/02688697.2017.1394444) | Population |
| Polster et al., 2017 (doi: 10.3171/2017.5.JNS17788) | Population |
| Gessler et al., 2017 (doi: 10.3171/2016.11.JNS161871) | Population |
| Coburger et al., 2014 (doi: 10.3171/2013.9.JNS122207) | Population |
| Ma et al., 2017 (doi: 10.1212/WNL.0000000000003902) | Population |
| Nam et al., 2017 (doi: 10.1371/journal.pone.0172793) | Population |
| Jean et al., 2017 (doi: 10.1016/j.jocn.2016.12.040) | Population |
| Samadani et al., 2015 (doi: 10.3171/2014.10.JNS14762) | Population |
| Mariotte et al., 2016 (doi: 10.1016/S2352-3026(16)30018-7) | Population |
| Labidi et al., 2015 (doi: 10.3171/2014.12.JNS141240) | Population |
| Molad et al., 2016 (doi: 10.1007/s11060-016-2291-x) | Population |
| Apra et al., 2015 (doi: 10.3171/2015.11.JNS151881) | Population |
| Zhang et al., 2017 (doi: 10.1007/s00415-016-8367-x) | Population |
| Hsu et al., 2016 (doi: 10.1155/2016/6205158) | Population |
| Bur et al., 2016 (doi:10.1001/jamaoto.2016.2807) | Population |
| Maldonado et al., 2016 (doi: 10.1016/j.jvsv.2016.05.003) | Population |
| Murthy et al., 2016 (doi: 10.1016/j.jstrokecerebrovasdis.2016.08.006) | Population |
| Kuhn et al., 2016 (doi: 10.1227/NEU.0000000000001378) | Population |
| Almekhlafi, 2016 (doi: 10.5144/0256-4947.2016.197) | Population |
| Suthar et al., 2016 (doi: 10.4103/1596-3519.176259) | Population |
| Liang et al., 2016 (doi: 10.1016/j.jvs.2015.12.047) | Population |
| Kennedy et al., 2016 (doi: 10.1016/j.jocn.2015.11.005) | Population |
| Farahmand et al., 2018 (doi: 10.1016/j.clineuro.2018.09.008) | Population |
| Nicolajsen et al., 2015 (doi: 10.1016/j.jvs.2015.06.223) | Population |
| Algethamy et al., 2015 (https://dx.doi.org/10.1017/cjn.2015.52) | Population |
| Huh et al., 2015 (doi: 10.1111/jgs.13298) | Population |
| Alonso-Martínez et al., 2014 (doi: 10.1016/j.medcli.2013.11.041) | Population |
| Moutzouris et al., 2014 (doi: 10.1111/jgs.13063) | Population |
| Kero et al., 2013 (doi: 10.1002/ijc.28385) | Population |
| Shah et al., 2013 (doi: 10.3109/02688697.2013.861387) | Population |
| Pósfai et al., 2013 (doi: 10.1016/j.jns.2013.10.016) | Population |
| Nieuwkamp et al., 2013 (doi: 10.1007/s00415-013-7192-8) | Population |
| Pongmoragot et al., 2013 (doi: 10.1161/JAHA.113.000372) | Population |
| Wierckx et al., 2013 (doi: 10.1530/EJE-13-0493) | Population |
| Salunke et al., 2013 (doi: 10.1016/j.clineuro.2013.06.003) | Population |
| Song et al., 2020 (doi: 10.1161/ATVBAHA.120.314269) | Population |
| Roubin et al., 2022 (doi: 10.1016/j.amjcard.2021.11.053) | Population |
| Bettag et al., 2021 (doi: 10.1007/s11060-021-03792-w) | Population |
| Abdelsalam et al., 2020 (doi: 10.1016/j.jstrokecerebrovasdis.2020.105299) | Population |
| Zhang et al., 2020 (doi: 10.1016/j.jstrokecerebrovasdis.2020.105026) | Population |
| Arai et al., 2020 (doi: 10.1007/s00701-020-04295-9) | Population |
| Aspberg et al., 2019 (doi: 10.1016/j.jstrokecerebrovasdis.2019.104560) | Population |
| Roux et al., 2022 (doi: 10.1007/s10143-021-01580-8) | Population |
| Brailovsky et al., 2019 (doi: 10.1016/j.jvsv.2019.10.014) | Population |
| Zhang et al., 2020 (doi: 10.1016/j.wneu.2019.12.063) | Population |
| Chethan et al., 2019 (doi: 10.1007/s12028-019-00835-z) | Population |
| Lee et al., 2019 (doi: 10.1007/s00415-019-09416-8) | Population |
| Adelborg et al., 2019 (doi: 10.1111/jth.14475) | Population |
| Díaz et al., 2019 (doi: 10.4067/s0034-98872019000200145) | Population |
| Kawano et al., 2019 (doi: 10.1016/j.jstrokecerebrovasdis.2018.10.012) | Population |
| Shoji et al., 2020 (doi: 10.2176/nmc.oa.2019-0208) | Population |
| Svensson et al., 2017 (doi: 10.1161/STROKEAHA.117.017849) | Population |
| Nguyen et al., 2017 (doi: 10.1016/j.wneu.2017.01.098) | Population |
| Plasencia-Martínez et al., 2016 (doi: 10.1016/j.ejrad.2016.12.010) | Population |
| Palazzo et al., 2016 (doi: 10.1161/STROKEAHA.116.015294) | Population |
| Corraini et al., 2016 (doi: 10.1016/j.thromres.2016.09.029) | Population |
| Shibahara et al., 2016 (doi: 10.1007/s11060-016-2085-1) | Population |
| Sjavik et al., 2016 (doi: 10.1016/j.wneu.2015.12.077) | Population |
| Safaee et al., 2014 (doi: 10.1016/j.clineuro.2014.01.021) | Population |
| Beynon et al., 2015 (doi: 10.1097/ANA.0000000000000104) | Population |
| Rahman et al., 2015 (doi: 10.1016/j.wneu.2015.02.016) | Population |
| Moussa and Mohamed, 2016 (doi: 10.1016/j.clineuro.2016.02.040) | Population |
| Dubinski et al., 2020 (doi: 10.1016/j.jocn.2020.09.059) | Population |
| Cerase et al., 2023 (doi: 10.1007/s10140-023-02115-y) | Population |
| Varela et al., 2023 (doi: 10.1016/j.ejso.2023.01.028) | Population |
| Munch et al., 2015 (doi: 10.1093/neuonc/nou312) | Population |
| Abt et al., 2014 (doi: 10.1016/j.jocn.2014.05.010) | Population |
| Thirunavu et al., 2021 (doi: 10.1016/j.wneu.2021.06.076) | Population |
| Barkley et al., 2020 (doi: 10.1016/j.wneu.2020.09.084) | Population |
| Zhang et al., 2021 (doi: 10.1016/j.jocn.2020.12.023) | Population |
| Petterson et al., 2015 (doi: 10.1016/j.thromres.2014.12.013) | Population |
| Dasenbrock et al., 2015 (doi: 10.3171/2015.10.FOCUS15386) | Population |
| Wei et al., 2019 (doi: 10.3389/fneur.2019.00579) | Population |
| Parikh et al., 2017 (doi: 10.1016/j.jstrokecerebrovasdis.2017.05.031) | Population |
| Zhang, Lin, Liu, 2023 (doi: 10.26355/eurrev_202304_32142) | Population |
| Wirsching et al., 2018 (doi: 10.1007/s11060-018-2996-0) | Population |
| Kimmell and Walter, 2014 (doi: 10.1007/s11060-014-1587-y) | Population |
| Nunno et al., 2019 (doi: 10.1016/j.wneu.2018.22.091) | Population |
| Sandblad et al., 2023 (doi: 10.1177/10760296231158368) | Population |
| Lee et al., 2019 (doi: 10.1016/j.wneu.2019.05.023) | Population (aneurysm) |
| https://www.jstage.jst.go.jp/article/circj/86/2/86_CJ-21-0631/_article | Population (atrial fibrillation) |
| Pastori et al., 2021 (doi: 10.1002/cncr.33470) | Population (atrial fibrillation, metastasis, other cancer site) |
| Osorio et al., 2023 (doi: 10.1227/neu.0000000000002404) | Population (benign) |
| https://dx.doi.org/10.4103/0028-3886.349613 | Population (benign) |
| Cross et al., 2022 (doi: 10.3390/curroncol29070390) | Population (benign) |
| Lee et al., 2022 (doi: 10.1016/j.wneu.2022.04.088) | Population (benign) |
| Huang et al., 2022 (doi: 10.1016/j.wneu.2022.03.080) | Population (benign) |
| Muliang et al., 2018 (doi: 10.1007/s11060-018-2952-z) | Population (benign) |
| Matthew et al., 2018 (doi: 10.1097/MAO.0000000000001806) | Population (benign) |
| Takeshima et al., 2017 (doi: 10.1016/j.wneu.2017.06.052) | Population (benign) |
| Quick-Weller et al., 2016 (10.1016/j.wneu.2016.04.130) | Population (benign) |
| Sluzewski et al., 2013 (doi: 10.3174/ajnr.A3311) | Population (benign) |
| Spirollari et al., 2022 (doi: 10.1016/j.wneu.2022.06.121) | Population (benign) |
| Wilhelmy et al., 2020 (doi: 10.1371/journal.pone.0238387) | Population (benign) |
| Eisenring et al., 2013 (doi: 10.1371/journal.pone.0079170) | Population (benign) |
| Lasica et al., 2022 (doi: 10.1371/journal.pone.0273189) | Population (benign) |
| Haeren et al., 2021 (doi: 10.1007/s11060-020-03693-4) | Population (benign) |
| Spinazzi et al., 2018 (doi: 10.1016/j.jocn.2018.09.018) | Population (benign) |
| Liu et al., 2019 (doi: 10.1002/cam4.2872) | Population (lymphoma) |
| Karhade et al., 2017 (doi: 10.1007/s11060-016-2262-2) | Population (meningioma) |
| Ikawa et al., 2023 (doi: 10.1186/s13014-023-02262-z) | Population (metastasis) |
| https://dx.doi.org/10.18071/isz.71.0178 | Population (metastasis) |
| Raisi-Estabragh et al., 2023 (doi: 10.1136/heartjnl-2022-321888) | Population (other cancer site) |
| Khanafer et al., 2023 (doi: 10.1016/j.neurad.2022.03.004) | Population (other cancer site) |
| Gon et al., 2020 (doi: 10.1016/j.jstrokecerebrovasdis.2020.104943) | Population (other cancer site) |
| Torre et al., 2020 (doi: 10.1016/j.jasc.2019.09.001) | Population (other cancer site) |
| Okura et al., 2018 (doi: 10.1007/s10147-018-1341-0) | Population (other cancer site) |
| Corley et al., 2019 (doi: 10.1016/j.amjcard.2018.11.008) | Population (other cancer site) |
| Shah et al., 2018 (doi: 10.1182/bloodadvances.2017010694) | Population (other cancer site) |
| Shin et al., 2016 (doi: 10.1007/s11060-016-2106-0) | Population (other cancer site) |
| Shayne et al., 2013 (doi: 10.1016/j.jgo.2013.05.005) | Population (other cancer site) |
| Kamphuisen et al., 2018 (doi: 10.1111/jth.14007) | Population (other cancer site) |
| Choi et al., 2023 (doi: 10.1186/s12883-023-03208-4) | Population (other cancer site) |
| Ardeshirrouhanifard et al., 2022 (doi: 10.1002/phar.2679) | Population (other cancer site) |
| Ording etv al., 2022 (doi: 10.1111/bjh.18060) | Population (other cancer site) |
| Sivakumar et al., 2021 (doi: 10.1002/onco.13897) | Population (other cancer site) |
| Merlino et al., 2021 (doi: 10.1038/s41598-021-91257-5) | Population (other cancer site) |
| Seystahl et al., 2021 (doi: 10.1007/s00415-021-10528-3) | Population (other cancer site) |
| Navi et al., 2021 (doi: 10.1002/ana.26129) | Population (other cancer site) |
| Yoo et al., 2021 (doi: 10.1161/STROKEAHA.120.032380) | Population (other cancer site) |
| Saito et al., 2021 (doi: 10.1038/s41598-021-83368-w) | Population (other cancer site) |
| Shen et al., 2020 (doi: 10.1097/MD.0000000000018779) | Population (other cancer site) |
| Atterman et al., 2020 (doi: 10.1093/europace/euz306) | Population (other cancer site) |
| Bang et al., 2019 (doi: 10.1161/STROKEAHA.119.026373) | Population (other cancer site) |
| Ha et al., 2019 (doi: 10.1161/JAHA.119.013215) | Population (other cancer site) |
| Fujinami et al., 2018 (doi: 10.1159/000491436) | Population (other cancer site) |
| Silvis et al., 2018 (doi: 10.1111/jth.13903) | Population (other cancer site) |
| Ahn et al., 2016 (doi: 10.1177/1076029615625826) | Population (other cancer site) |
| Scharzbach et al., 2015 (doi: 10.1159/000439549) | Population (other cancer site) |
| Navi et al., 2014 (doi: 10.1212/WNL.0000000000000539) | Population (other cancer site) |
| Capellari et al., 2013 (doi: 10.1016/j.jns.2012.12.008) | Population (other cancer site) |
| Sorretino et al., 2014 (doi: 10.1097/MPH.0000000000000144.) | Population (other cancer site) |
| https://dx.doi.org/10.3109/14767058.2015.1009439 | Population (pregnancy and cancer) |
| Kassicieh et al., 2022 (doi: 10.3171/2022.9.FOCUS22402) | Population (primary cancer site not specified) |
| Otite et al., 2022 (doi: 10.1016/j.jstrokecerebrovasdis.2022.106818) | Population (primary cancer site not specified) |
| Szlachetka et al., 2022 (doi: 10.1007/s13760-021-01752-9) | Population (primary cancer site not specified) |
| Menichelli et al., 2021 (doi: 10.1016/j.pcad.2021.04.004) | Population (primary cancer site not specified) |
| Pristavu et al., 2021 (doi: 10.2478/raon-2021-0014) | Population (primary cancer site not specified) |
| Loggini et al., 2021 (doi: 10.1016/j.jstrokecerebrovasdis.2020.105584) | Population (primary cancer site not specified) |
| Hug et al., 2021 (doi: 10.1093%2Fneuros%2Fnyaa335) | Population (primary cancer site not specified) |
| Gusdon et al., 2021 (doi: 10.1161/STROKEAHA.119.027085) | Population (primary cancer site not specified) |
| Cacho-Díaz et al. 2018 (doi: 10.1007/s11060-018-2982-6) | Population (primary cancer site not specified) |
| Green et al., 2018 (doi: 10.3324/haematol.2017.182220) | Population (primary cancer site not specified) |
| Elmariah et al., 2018 (doi: 10.1161/CIRCINTERVENTIONS.117.005795) | Population (primary cancer site not specified) |
| Nochioka et al., 2022 (doi: doi: 10.1002/ehf2.13941) | Population (primary cancer site not specified) |
| Jeon et al., 2020 (doi: 10.1136/neurintsurg-2020-016144) | Population (primary cancer site not specified) |
| Balabhadra et al., 2020 (doi: 10.1001/jamanetworkopen.2020.11079) | Population (primary cancer site not specified) |
| Chatterjee et al., 2019 (doi: 10.1016/j.jstrokecerebrovasdis.2019.05.009) | Population (primary cancer site not specified) |
| Park et al., 2019 (doi: 10.1002/ana.25495) | Population (primary cancer site not specified) |
| Weeda et al., 2018 (doi: 10.1177/1747493018778135) | Population (primary cancer site not specified) |
| Barrios et al., 2017 (doi: 10.1371/journal.pone.0187648) | Population (primary cancer site not specified) |
| Sprugel et al., 2017 (doi: 10.1159/000479075) | Population (primary cancer site not specified) |
| Melloni et al., 2017 (doi: 10.1093/ehjqcco/qcx004) | Population (primary cancer site not specified) |
| Thalin et al., 2016 (doi: 10.1016/j.thromres.2016.01.009) | Population (primary cancer site not specified) |
| Lee et al., 2015 (doi: 10.1016/j.ijcard.2015.10.166) | Population (primary cancer site not specified) |
| Liu et al., 2014 (doi: 10.1111/cns.12227) | Population (primary cancer site not specified) |
| http://dx.doi.org/10.4070/kcj.2017.0328 | Population (primary cancer site not specified) |
| Andersen and Olsen, 2018 (doi: 10.1161/STROKEAHA.118.021373) | Population (primary cancer site not specified) |
| Englisch et al., 2022 (doi: 10.3390/ijms232415770) | Population (primary cancer site not specified) |
| Leader et al., 2022 (doi: 10.1111/jth.15600) | Population/Outcome |
| Wang, Kim and Kim, 2021 (doi: 10.1016/j.mayocp.2020.05.045) | Population/Outcome |
| Leclerc et al., 2022 (doi: 10.1016/j.neuchi.2022.02.008) | Population/Outcome |
| Salah and Shalaby, 2022 (doi: 10.25259/SNI_1131_2022) | Population/Outcome |
| Feng et al., 2018 (doi: 10.1097/MD.0000000000010840) | Population/Outcome |
| Dandachi et al., 2019 (doi: 10.1007/s10552-019-01144-8) | Population/Outcome |
| Press et al., 2019 (doi: 10.1093/neuros/nyy436) | Population/Outcome |
| Conner et al., 2018 (doi: 10.1016/j.jocn.2018.09.001) | Population/Outcome |
| Akiyama et al., 2018 (doi: 10.1016/j.wneu.2018.02.070) | Population/Outcome |
| Alkhalid et al., 2017 (doi: 10.1016/j.clineuro.2017.10.018) | Population/Outcome |
| Barraco et al., 2016 (doi: 10.1016/j.neurol.2016.09.010) | Population/Outcome |
| Schebesch et al., 2015 (doi: 10.1016/j.clineuro.2015.04.006) | Population/Outcome |
| Klinger et al., 2015 (doi: 10.1016/j.wneu.2015.04.033) | Population/Outcome |
| Kaley et al., 2015 (doi: 10.1093/neuonc/nou148) | Population/Outcome |
| Shakal and Mokbel, 2014 (doi: 10.1055/s-0032-1325633) | Population/Outcome |
| Takami et al., 2022 (doi: 10.1016/j.wneu.2022.08.102) | Population/Outcome |
| Lin et al., 2022 (doi: 10.1155/2022/9367919) | Population/Outcome |
| Hosainey et al., 2021 (doi: 10.1007/s10143-021-01648-5) | Population/Outcome |
| Takami et al., 2020 (doi: 10.3171/2020.4.JNS20378) | Population/Outcome |
| Aliouat et al., 2022 (doi: 10.1007/s11060-022-04069-6) | Population/Outcome |
| Zanello et al., 2021 (doi: 10.1016/j.wneu.2020.12.127) | Population/Outcome |
| Richardson et al., 2019 (doi: 10.1016/j.wneu.2019.03.004) | Population/Outcome |
| Ramin et al., 2018 (doi: 10.3171/2018.4.JNS18375) | Population/Outcome |
| De la Garza-Ramos et al., 2016 (doi: 10.1016/j.clineuro.2015.11.005) | Population/Outcome |
| Wirsching et al., 2016 (doi: 10.1093/neuonc/nov303) | Population/Outcome |
| Lund et al., 2015 (doi: 10.1016/j.thromres.2015.09.001) | Population/Outcome |
| Yuen et al., 2014 (doi: 10.1159/000360862) | Population/Outcome |
| Hawasli et al., 2013 (doi: 10.1227/01.neu.0000430310.63702.3e) | Population/Outcome |
| Byun et al., 2019 (doi: 10.1016/j.thromres.2019.10.002) | Population/Outcome |
| Xu et al., 2020 (doi: 10.1097/SCS.0000000000005866) | Population/Outcome |
| Gazal et al., 2020 (doi: 10.1159/000512241) | Population/Outcome |
| Sarid et al., 2021 (doi: 10.1007/s11060-020-03654-x) | Population/Outcome |
| Callovini et al., 2021 (doi: 10.1016/j.wneu.2021.01.035) | Population/Outcome |
| Saito, Wages & Schiff, 2021 (doi: 10.1007/s11060-021-03791-x) | Population/Outcome |
| Ong et al., 2016 (doi: 10.1016/j.neurol.2016.09.010) | Population/Outcome |
| Raper et al., 2017 (doi: 10.1016/j.wneu.2016.11.084) | Population/Outcome |
| Chen et al., 2021 (doi: 0.1007/s00228-021-03132-x) | Type of study |
| Isenberg et al., 2021 (doi: 10.1111/acem.14205) | Type of study |
| Saiegh et al., 2020 (doi: 10.1016/j.clineuro.2020.106121) | Type of study |
| Benyaich et al., 2020 (doi: 10.1016/j.wneu.2020.04.039) | Type of study |
| Cheng et al., 2022 (doi: 10.1080/00207454.2020.1858824) | Type of study |
| Sethi and Reddy, 2018 (doi: 10.1080/10428194.2018.1466296) | Type of study |
| Brawanski et al., 2020 (doi: 10.23736/S0390-5616.16.03859-5) | Type of study |
| Orlev et al., 2020 (doi: 10.1093/ons/opz396) | Type of study |
| Cavallari et al., 2020 (doi: 10.1055/s-0039-3400300) | Type of study |
| Dusart et al., 2019 (doi: 10.1016/j.celrep.2019.09.088) | Type of study |
| Cortez et al., 2020 (doi: 10.1136/bcr-2020-235662) | Type of study |
| Joseph et al., 2017 (doi: 10.1016/j.jocn.2016.12.046) | Type of study |
| Hempel et al., 2019 (doi: 10.1007/s00062-018-0676-2) | Type of study |
| Jaeckle et al., 2020 (doi: 10.1007/s11060-019-03194-z) | Type of study |
| Kohli, Chaturvedi, 2019 (doi: 10.1055/s-0039-1683416) | Type of study |
| Song et al., 2019 (doi: 10.1016/j.radonc.2018.12.019) | Type of study |
| Chen et al., 2019 (doi: 10.1093/ehjqcco/qcy040) | Type of study |
| Grasso et al., 2014 (doi: 10.12659/MSM.890583) | Type of study |
| Wang et al., 2015 (doi: 10.1097/NRL.0000000000000027) | Type of study |
| Halalmeh et al., 2023 (doi: 10.1016/j.wneu.2022.10.113) | Type of study |
| Totzeck et al., 2018 (doi: 10.1177/2047487318755193) | Type of study |
| Koike et al., 2017 (doi: 10.2217/fca-2017-0063) | Type of study |
| Mohan et al., 2016 (doi: 10.2217/cns-2016-0003) | Type of study |
| Nagahisa et al., 2013 (doi: 10.1007/s10143-013-0467-9) | Type of study |
| Diaz and Schiff, 2022 (doi: 10.1097/CCO.0000000000000875) | Type of study |
| Mosconi et al., 2022 (doi: 10.1007/s10072-022-06026-7) | Type of study |
| Sevestre and Soudet, 2020 (doi: 10.1016/S2542-4513(20)30513-7) | Type of study |
| Muster and Gary, 2020 (doi: 10.3390/cancers12061354) | Type of study |
| Geyik et al., 2021 (doi: 10.5137/1019-5149.JTN.33015-20.2) | Type of study |
| Riedl et al., 2016 (doi; 10.1016/S0049-3848(16)30196-7) | Type of study |
| Denas et al., 2015 (doi: 10.1016/j.thromres.2015.06.011) | Type of study |
| Rinaldo et al., 2019 (doi: 10.3171/2018.7.JNS181175) | Type of study |
| Al Shaikh et al., 2021 (doi: 10.1016/j.jstrokecerebrovasdis.2021.106019) | Type of study |
| Wuthrick et al., 2014 (doi: 10.1016/j.ijrobp.2014.05.034) | Type of study |
| Keil et al., 2017 (doi: 10.3174/ajnr.A5313) | Type of study |
| Shamsdin et al., 2019 (doi: 10.31557/APJCP.2019.20.10.2883) | Type of study (case control) |
| Gritsch et al., 2022 (doi: 10.1097/NRL.0000000000000402) | Type of study (case report) |
| Sizdahkhani, Magill, McDermott, 2017 (doi: 10.1016/j.wneu.2017.07.067) | Type of study (case report) |
| Alexandrescu et al., 2015 (doi: 10.1111/neup.12175) | Type of study (case report) |
| Dixit et al., 2013 (doi: 10.1007/s13760-013-0177-7) | Type of study (case report) |
| Adeleye and Ogun, 2016 (doi: 10.1016/j.wneu.2016.01.069) | Type of study (case report) |
| Lampert et al., 2017 (doi: 10.1136/bcr-2017-221578) | Type of study (case report) |
| Cai et al., 2014 (doi: 10.1186/1477-7819-12-110) | Type of study (case report) |
| Tomás-Biosca et al., 2022 (doi: 10.1097/ANA.0000000000000753) | Type of study (letter to editor) |
| Al Megren et al., 2017 (https://pubmed.ncbi.nlm.nih.gov/28624717/) | Other |
| Gazzeri et al., 2018 (https://pubmed.ncbi.nlm.nih.gov/28439721/) | Other |
| Jarvis et al., 2020 (https://pubmed.ncbi.nlm.nih.gov/32942082/) | Other |
| Mahajan et al., 2022 (https://pubmed.ncbi.nlm.nih.gov/34649273/) | Other |
| Mandel et al., 2021 (https://pubmed.ncbi.nlm.nih.gov/34146775/) | Other |
| Motoo et al., 2019 (https://www.ncbi.nlm.nih.gov/pmc/articles/PMC6923818/) | Other |
| Nabi et al., 2016 (https://pubmed.ncbi.nlm.nih.gov/26547860/) | Other |
| Posch et al., 2020 (https://pubmed.ncbi.nlm.nih.gov/32073229/) | Other |
| Qdaisat et al., 2020 (https://pubmed.ncbi.nlm.nih.gov/32376060/) | Other |
| Reitter et al., 2016 (https://pubmed.ncbi.nlm.nih.gov/26662117/) | Other |
| Reidl et al., 2014 (https://www.ncbi.nlm.nih.gov/pmc/articles/PMC4210186/) | Other |
| Smith et al., 2014 (https://pubmed.ncbi.nlm.nih.gov/25062669/) | Other |
| Smith et al., 2015 (https://pubmed.ncbi.nlm.nih.gov/25533212/) | Other |
| Zhang et al., 2022 (https://www.frontiersin.org/journals/oncology/articles/10.3389/fonc.2022.898873/full) | Other |
| Edwin et al., 2016 (https://pubmed.ncbi.nlm.nih.gov/26657302/) | Other |
| Kapteijn et al., 2023 (https://pubmed.ncbi.nlm.nih.gov/36435047/) | Other |
| Riedl et al., 2017 (https://pubmed.ncbi.nlm.nih.gov/28073783/) | Other |
| Shi et al., 2021 (https://pubmed.ncbi.nlm.nih.gov/34739883/) | Other |
