# Supplementary material for: Incidence of venous thromboembolism and bleeding in patients with malignant central nervous system neoplasm: Systematic review and meta-analysis
Source: PLoS One. 2024 Jun 20;19(6):e0304682. doi: 10.1371/journal.pone.0304682 (PMC11189257; doi:10.1371/journal.pone.0304682)
Supplement: S3 Table — (DOCX) [file pone.0304682.s008.docx]

S3 Table. Conflict of interest and funding reported in the included studies.

| Author, yr | Conflict of interest | Funding |
| --- | --- | --- |
| Auer et al., 2017 | None. | Not informed. |
| Barbaro et al., 2022 | None. | None. |
| Bruhns et al., 2018 | None. | None. |
| Carney et al., 2018 | None. | Not informed. |
| Diaz et al., 2021 | None. | None. |
| Ebeling et al., 2018 | None. | Not informed. |
| Eisele et al., 2021 | Informed. | Informed. |
| Ening et al., 2014 | Not informed. | Not informed. |
| Fisher et al., 2014 | Not informed. | Informed. |
| Gazzeri et al., 2017 | None. | None. |
| Helmi et al., 2019 | Informed. | Not informed. |
| Huang et al., 2022 | None. | Informed. |
| Jo et al., 2022 | None. | None. |
| Kaptein et al., 2021 | Informed. | Not informed. |
| Kaye et al., 2023 | None. | None. |
| Khoury et al., 2016 | None. | Informed. |
| Lee et al., 2019 | Informed. | Informed. |
| Lee et al., 2022 | None. | None. |
| Lim et al., 2018 | None. | None. |
| Liu et al., 2019 | None. | Not informed. |
| Liu et al., 2023 | None. | Informed. |
| Mantia et al., 2017 | Informed. | Not informed. |
| McGahan et al., 2017 | None. | Informed. |
| Missios al., 2015 | None. | Not informed. |
| Nakano et al., 2018 | None. | None. |
| Nazari et al., 2020 | Informed. | Informed. |
| Park et al., 2021 | Informed. | None. |
| Rahman et al., 2015 | None. | None. |
| Rinaldo et al., 2019 | None. | Not informed. |
| Seidel et al., 2013 | None. | Informed. |
| Senders et al., 2018 | None. | Not informed. |
| Shi et al., 2020 | None. | Informed. |
| Streiff et al., 2015 | Informed. | Informed. |
| Thaler et al., 2013 | None. | Informed. |
| Unruh et al., 2016 | Not informed. | Informed. |
| Zhang et al., 2023 | None. | Informed. |
| Zhou et al., 2022 | None. | Informed. |
