# Supplementary material for: Incidence of venous thromboembolism and bleeding in patients with malignant central nervous system neoplasm: Systematic review and meta-analysis
Source: PLoS One. 2024 Jun 20;19(6):e0304682. doi: 10.1371/journal.pone.0304682 (PMC11189257; doi:10.1371/journal.pone.0304682)
Supplement: Attachment — Submitted filename: Response to Reviewers.docx [file pone.0304682.s010.docx]

São Paulo, 14th, May 2024

Dear Academic Editor,

Omar A. Almohammed, Ph.D.

Thank you for giving us the opportunity to resubmit the manuscript titled “Incidence of venous thromboembolism and bleeding in patients with malignant central nervous system neoplasm: systematic review and meta-analysis”. We hope the manuscript after careful revisions meet your standards to be accepted for publication. All modifications in the manuscript have been with tracked changes.

We ensured that the bibliographic references were reviewed and adjusted to meet the journal's guidelines. Additionally, we identified an erratum in reference 55 (page 35, line 783-787):

*“Wu S, Lv M, Chen J, Jiang S, Chen M, Fang Z, et al. Direct oral anticoagulants for venous thromboembolism in cancer patients: a systematic review and network meta-analysis. Support Care Cancer. 2022 Dec;30(12):10407–20; discussion 10416-18. Erratum in: Support Care Cancer. 2023 Jun 3;31(6):373. doi: 10.1007/s00520-023-07851-y.”*

However, the text of the discussion did not contain any errors, as the information was discussed with the current version. We also emphasize that this article was not included in those listed for the meta-analysis.

Concerning the reviewers' comments, we noted that Review 1 indicated item 6 raised some questions.

We sincerely appreciate all comments and suggestions provided.

On behalf of the authors

Viviane Cordeiro Veiga, MD, Ph.D

--------------------------------------------------------- Reviewer #1--------------------------------------------------

Dear Reviewer,

Thank you for your precious time in reviewing our paper and providing valuable comments.

6. Review Comments to the Author

Please use the space provided to explain your answers to the questions above. You may also include additional comments for the author, including concerns about dual publication, research ethics, or publication ethics. (Please upload your review as an attachment if it exceeds 20,000 characters)

Reviewer #1:

Dear Authors,

Firstly, congratulations on your remarkable work! However, upon review, it appears that some language edits are necessary throughout the document. Additionally, we noticed that your conclusions seem to mirror your results, which might benefit from further refinement to avoid redundancy.

Answer: Thank you for your consideration and we have changed the paragraphs in abstract, discussion, and conclusion:

Abstract – page 3, lines 51 – 54.

**Before** “The pooled incidence was 13.68% and 11.60% for VTE and bleeding, respectively. Being older than 60 years or having GBM diagnosis led to pooled incidence of 32.27% and 16.10% for VTE.”

**After** “Our study showed variability in the pooled incidence for both overall events and subgroup analyses. It was highlighted that individuals over 60 years old or diagnosed with GBM had a higher pooled incidence of VTE among those with overall CNS malignancies.”

Discussion – page 24, lines 436 – 437.

**Before** “In the present study, the pooled incidence of VTE was 13.68%. The incidence was similar to values previously reported (54)”.

**After** “In the present study, the pooled incidence of VTE was similar to values previously reported (54).

Discussion – page 25, lines 452 – 452.

**Before** “For GBM, the pooled incidence was 16.10% (95%CI 10.52; 22.57) for VTE.”

**After** “For GBM, the pooled incidence of VTE was higher than that of overall CNS malignant neoplasms.”

Conclusion – page 29, lines 559 – 565.

**Before** “The pooled incidence was 13.68% for VTE. Similarly, the incidence pooled of bleeding was 11.60%. Subgroup analysis showed that being older than 60 years or having GBM diagnosis presented higher pooled incidence values in comparison to overall CNS malignant neoplasm. Further studies from low and lower-middle income countries should be encouraged.”

**After** *“According to this research, the pooled incidence showed variability across all analyses and their subgroups for both events. Subgroup analysis showed that being older than 60 years or having GBM diagnosis presented higher pooled incidence values in comparison to overall CNS malignant neoplasm. In addition to the sensitivity analysis, when considering the outlier criterion, it's noted a higher pooled incidence among GBM, mirroring findings in the literature. Further studies from low and lower-middle income countries should be encouraged.”*
